# Evaluation of Heterologous Biosynthetic Pathways for Methanol-Based 5-Aminovalerate Production by Thermophilic *Bacillus methanolicus*

**DOI:** 10.3389/fbioe.2021.686319

**Published:** 2021-06-28

**Authors:** Luciana Fernandes Brito, Marta Irla, Ingemar Nærdal, Simone Balzer Le, Baudoin Delépine, Stéphanie Heux, Trygve Brautaset

**Affiliations:** ^1^Department of Biotechnology and Food Science, Norwegian University of Science and Technology, Trondheim, Norway; ^2^Department of Biotechnology and Nanomedicine, SINTEF Industry, Trondheim, Norway; ^3^Toulouse Biotechnology Institute, Université de Toulouse, CNRS, INRA, INSA, Toulouse, France

**Keywords:** *Bacillus methanolicus*, thermophile, methanol, 5-aminovalerate, alternative feedstock

## Abstract

The use of methanol as carbon source for biotechnological processes has recently attracted great interest due to its relatively low price, high abundance, high purity, and the fact that it is a non-food raw material. In this study, methanol-based production of 5-aminovalerate (5AVA) was established using recombinant *Bacillus methanolicus* strains. 5AVA is a building block of polyamides and a candidate to become the C5 platform chemical for the production of, among others, δ-valerolactam, 5-hydroxy-valerate, glutarate, and 1,5-pentanediol. In this study, we test five different 5AVA biosynthesis pathways, whereof two directly convert L-lysine to 5AVA and three use cadaverine as an intermediate. The conversion of L-lysine to 5AVA employs lysine 2-monooxygenase (DavB) and 5-aminovaleramidase (DavA), encoded by the well-known *Pseudomonas putida* cluster *davBA*, among others, or lysine α-oxidase (RaiP) in the presence of hydrogen peroxide. Cadaverine is converted either to γ-glutamine-cadaverine by glutamine synthetase (SpuI) or to 5-aminopentanal through activity of putrescine oxidase (Puo) or putrescine transaminase (PatA). Our efforts resulted in proof-of-concept 5AVA production from methanol at 50°C, enabled by two pathways out of the five tested with the highest titer of 0.02 g l^–1^. To our knowledge, this is the first report of 5AVA production from methanol in methylotrophic bacteria, and the recombinant strains and knowledge generated should represent a valuable basis for further improved 5AVA production from methanol.

## Introduction

The worldwide amino acid market is progressively growing at 5.6% annual rate and is estimated to reach US$25.6 billion by 2022, with amino acids used for animal feed production being its largest component (Wendisch, 2020). The growing demand for amino acid supply confronts the biotechnological industry with an unprecedented challenge of identifying suitable feedstocks, especially in terms of replacing sugars and agricultural products, use whereof deteriorates food supply and threatens biodiversity (Cotton et al., 2020). Methanol, together with other one-carbon (C1) compounds, is considered a very promising substitute for feedstock that are conventionally used in biotechnological processes. The major advantages of using methanol as carbon source are its low production cost (e.g., methanol from steam reforming of methane), ease of transport and storage, and complete miscibility that bypasses the mass transfer barrier and potentially supports improvement in microbial productivities. However, what seems to cause a considerable difficulty in propagation of methanol as biotechnological feedstock is the limited selection of microorganisms capable to be used as their carbon and energy source. One of the compelling candidates to become a workhorse for the methanol-based production of amino acids is *Bacillus methanolicus*, a thermophilic methylotroph isolated from freshwater marsh soil by Schendel et al. (1990). The wild-type strain MGA3 naturally overproduces L-glutamate in methanol-controlled fed-batch fermentations with volumetric titers reaching up to 60 g l^–1^ (Heggeset et al., 2012; Table 1). Furthermore, thanks to recent developments in the toolbox for gene overexpression, it was engineered for production of different amino acid derivatives such as γ-aminobutyric acid and cadaverine (Nærdal et al., 2015; Irla et al., 2017; Table 1). MGA3 produces 0.4 g l^–1^ of L-lysine in high cell density fed-batch fermentations (Brautaset et al., 2010; Table 1); this titer was improved nearly 30-fold up to 11 g l^–1^ by plasmid-based overexpression of a gene coding for aspartokinase, a key enzyme controlling the synthesis of aspartate-derived amino acids (Jakobsen et al., 2009). Through application of a classical mutagenesis technique, a derivative of *B. methanolicus* MGA3 (M168-20) was constructed, which produces 11 g l^–1^ of L-lysine in high cell density methanol-controlled fed-batch fermentations (Brautaset et al., 2010); the L-lysine overproduction being caused among others by mutation in the *hom-*1 gene coding for homoserine dehydrogenase (Hom) and in the putative lysine 2,3-aminomutase gene (locus tag BMMGA3_02505). The mutation in *hom*-1 leads to the loss of catalytic activity of homoserine dehydrogenase and redirection of metabolic flux toward the L-lysine pathway and therefore its accumulation (Nærdal et al., 2011, 2017).

**TABLE 1:** Comparison of the 5AVA production by different engineered microbial strains and production of amino acids by *B. methanolicus*.

**Organism**	**Approach**	**5AVA titer [g l**^–^**^1^]**	**References**
*Pseudomonas putida* KT2440	DavBA-based biocatalytic production of 5AVA from 30 g l^–1^ L-lysine	20.80	Liu et al., 2014
*Corynebacterium glutamicum*	Heterologous expression of *davBA*; sugar-based fed-batch fermentation	33.10	Shin et al., 2016
		28.00	Rohles et al., 2016
		39.93	Joo et al., 2017
	Heterologous expression of *ldcC* and *patAD*; shake flask fermentation	5.10	Jorge et al., 2017
	Heterologous expression of *puo* and *patD*, deletion of *gabTD*; microbioreactor fermentation	3.70	Haupka et al., 2020
*Escherichia coli*	Heterologous expression of *davBA* and deletion of *cadA*; glucose-based shaking flasks fermentation	0.86	Adkins et al., 2013
	Heterologous expression of *davBA*; sugar-based fermentation; 10 g l^–1^ lysine provided	3.60	Park et al., 2013
	Heterologous expression of *davBA*; sugar-based fed-batch fermentation	0.50	Park et al., 2013
	Heterologous expression of *davBA*; glucose-based fed- batch fermentation; 120 g l^–1^ L-lysine provided	90.59	Park et al., 2014
	Heterologous expression of *davBA*; fed-batch whole-cell bioconversion of L-lysine maintained at 120 g l^–1^	240.70	Wang et al., 2016
	Heterologous expression of *raiP*; whole-cell bioconversion; addition of 4% ethanol, 10 mM H_2_O_2_ and 100 g l^–1^ lysine	29.12	Cheng et al., 2018
	Heterologous expression of *raiP*; whole-cell bioconversion; 4% ethanol pretreatment, 10 mM H_2_O_2_ and 100 g l^–1^ lysine	50.62	Cheng et al., 2020

**Organism**	**Product in methanol-controlled fed-batch fermentation**	**Titer [g L**^–^**^1^]**	**References**

*Bacillus methanolicus*	L-Glutamate	60.00	Heggeset et al., 2012
	L-Lysine	11.00	Brautaset et al., 2010
	γ-Aminobutyric acid	9.00	Irla et al., 2017
	Cadaverine	11.30	Nærdal et al., 2015

5-Aminovalerate (5AVA) is a product of L-lysine degradation, and it is mainly synthesized in a two-step process catalyzed by a lysine monooxygenase (DavB) and a δ-aminovaleramide amidohydrolase (DavA) (Revelles et al., 2005). 5AVA is a non-proteogenic five-carbon amino acid that could potentially be used as building block for producing biobased polyamides (Adkins et al., 2013; Park et al., 2014; Wendisch et al., 2018). It is also a promising precursor for plasticizers and chemicals that are intermediates for bioplastic preparation: δ-valerolactam (Chae et al., 2017), 5-hydroxy-valerate (Sohn et al., 2021), glutarate (Adkins et al., 2013; Pérez-García et al., 2018), and 1,5-pentanediol (Cen et al., 2021). As summarized in Table 1, diverse approaches have been made at the establishment of microbial 5AVA production. *Pseudomonas putida* KT2440, which possesses *davBA* in its genome, can synthesize 20.8 g l^–1^ 5AVA from 30 g l^–1^ L-lysine in 12 h (Liu et al., 2014). Production of 5AVA was established in *Corynebacterium glutamicum* by heterologous overexpression of the DavB- and DavA-encoding genes (*davBA*) from *P. putida* with a final titer up to 39.9 g l^–1^ in a sugar-based fed-batch fermentation (Rohles et al., 2016; Shin et al., 2016; Joo et al., 2017). 5AVA can be also produced in a process of bioconversion of L-lysine supplemented to the growth medium with molar yields of up to 0.942 achieved by *Escherichia coli* strains overproducing DavBA (Park et al., 2014; Wang et al., 2016). Moreover, when the recombinant *E. coli* strain expressing *davAB* genes was cultured in a medium containing 20 g l^–1^ glucose and 10 g l^–1^ L-lysine, 3.6 g l^–1^ 5AVA was produced, representing a molar yield of 0.45 (Park et al., 2013). Disruption of native lysine decarboxylase (CadA and LdcC) activity in *E. coli* strains overexpressing *davBA* limited cadaverine by-product formation, enabling increased accumulation of L-lysine following 5AVA production, with 5AVA yield of 0.86 g l^–1^ in glucose-based shaking flask fermentation (Adkins et al., 2013). Furthermore, Cheng et al. (2018) reported that the oxidative decarboxylation of L-lysine catalyzed by a L-lysine α-oxidase (RaiP) from *Scomber japonicus* led to 5AVA production. The production of RaiP was enhanced by the addition of 4% (v/v) ethanol and 10 mM H_2_O_2_, which increased the 5AVA titer to 29.12 g l^–1^ by an *E. coli* host strain in a fed-batch fermentation (Cheng et al., 2018). Recently, in a similar L-lysine bioconversion strategy, an *E. coli* whole-cell catalyst producing RaiP was developed, converting 100 g l^–1^ of L-lysine hydrochloride to 50.62 g l^–1^ 5AVA representing a molar yield of 0.84 (Cheng et al., 2020).

Recent efforts have employed novel metabolic routes toward 5AVA. In *Pseudomonas aeruginosa* PAO1, the set of enzymes composed of glutamylpolyamine synthetase, polyamine:pyruvate transaminase, aldehyde dehydrogenase, and glutamine amidotransferase is essential for the degradation of diamines through the γ-glutamylation pathway (Yao et al., 2011), which may lead to 5AVA production when cadaverine is degraded (Luengo and Olivera, 2020). Jorge et al. (2017) established a three-step 5AVA biosynthesis pathway consisting of the conversion of L-lysine to cadaverine by the activity of the enzyme LdcC, followed by cadaverine conversion to 5AVA through consecutive transamination, by a putrescine transaminase (PatA), and oxidation by a PatD. The heterologous overexpression of the genes *ldcC*, *patA*, and *patD* led to 5AVA production to a final titer of 5.1 g l^–1^ by an engineered *C. glutamicum* strain in a shake flask fermentation (Jorge et al., 2017). This pathway has served as basis for the establishment of a new three-step pathway toward 5AVA using the monooxygenase putrescine oxidase (Puo), which catalyzes the oxidative deamination of cadaverine, instead of PatA (Haupka et al., 2020).

**FIGURE 1:**
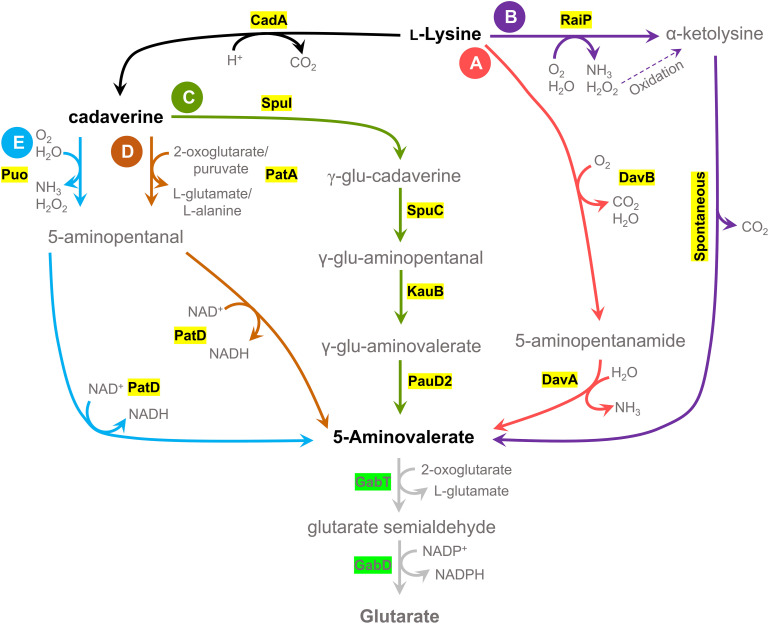
Schematic view of five 5AVA biosynthesis pathways and a 5AVA degradation pathway. Five different pathways for potential 5AVA production in *Bacillus methanolicus* were tested; two pathways have L-lysine as precursor, and three pathways have cadaverine as an intermediate metabolite, obtained by conversion of L-lysine by a lysine decarboxylase (CadA). **(A)** DavBA pathway: L-lysine conversion to 5AVA by lysine 2-monooxygenase (DavB) and 5-aminovaleramidase (DavA). **(B)** RaiP pathway: conversion of L-lysine to α-ketolysine by a L-lysine α-oxidase (RaiP) and spontaneous decarboxylation of α-ketolysine in the presence of hydrogen peroxide. **(C)** SpuI pathway: cadaverine to γ-glutamine-cadaverine (γ-glu-cadaverine) by glutamylpolyamine synthetase (SpuI), with subsequent activity of polyamine:pyruvate transaminase (SpuC), aldehyde dehydrogenase (KauB), and glutamine amidotransferase class I (PauD2); γ-glu-aminopentanal: γ-glutamine-aminopentanal, γ-glu-aminovalerate: γ-glutamine-aminovalerate. **(D)** PatA pathway: cadaverine to 5-aminopentanal through activity of putrescine aminase (PatA) and 5-aminopentanal conversion to 5AVA by 5-aminopentanal dehydrogenase (PatD). **(E)** Puo pathway: cadaverine to 5-aminopentanal through activity of putrescine oxidase (Puo), followed by 5AVA formation by PatD. 5AVA is degraded to glutarate by GABAse activity, a combination of γ-aminobutyrate aminotransferase (GabT) and succinate semialdehyde dehydrogenase (GabD), although this activity was not found in *B. methanolicus* (Irla et al., 2017).

Critical factors that can affect 5AVA accumulation in a production host are the presence of a native 5AVA degradation pathway in its genome and the end product-related inhibition. In some bacterial species, such as *P. putida* KT2440, *Pseudomonas syringae*, *Pseudomonas stutzeri*, and *C. glutamicum*, 5AVA is degraded by a GABAse (Figure 1), composed of two enzymes γ-aminobutyric acid aminotransferase (GabT) and succinic semialdehyde dehydrogenase (GabD) (Park et al., 2013; Rohles et al., 2016; Pérez-García et al., 2018); for example, GABAse from *Pseudomonas fluorescens* KCCM 12537 retains 47.7% activity when 5AVA is used as its substrate in comparison to when GABA is used (So et al., 2013). Based on the previous research, *B. methanolicus* seems a feasible candidate for 5AVA production because it does not possess the necessary genetic background for GABAse-based 5AVA degradation, lacking the *gabT* gene in its genome (Irla et al., 2017). It was reported that 5AVA does not supports growth of *B. methanolicus* neither as sole carbon source nor as sole nitrogen source (Haupka et al., 2021). However, *B. methanolicus* displays low tolerance to 5AVA, with growth being impaired by addition of 1.17 g l^–1^ 5AVA to the culture broth (Haupka et al., 2021).

Even though the application of diverse 5AVA biosynthetic pathways has led to significant improvement in titers and yields of 5AVA production in bacterial hosts, the most efficient processes rely on raw materials that contain sugar and/or agricultural products. Addressing shortages of global resources and food requires a replacement of the current mode of industrial biotechnology, which results in the need for novel biosynthetic pathways that utilize alternative raw materials such as methanol. Hence, in the present study we have selected five different pathways to establish methanol-based 5AVA production in the methylotrophic bacterium *B. methanolicus*. For two of the five pathways, proof-of-principle 5AVA production was achieved and our results should represent a valuable basis of knowledge and strains for further improved 5AVA production from methanol at 50°C.

## Materials and Methods

### Retrosynthesis Analysis

Retrosynthesis analysis was conducted with RetroPath 2 (Delépine et al., 2018) (v6) and RetroRules (Duigou et al., 2019) (1.0.2, with hydrogens, in reversed direction) that translated reactions from MetaNetX (Moretti et al., 2016) into reaction rules, and KNIME (3.6.1). The “source” used in this analysis was 5AVA [InChI = 1S/C5H11NO2/c6-4-2-1-3-5(7)8/h1-4,6H2,(H,7,8)], and “sink” was the set of all metabolites from *E. coli* genome-scale model iJO1366 (Orth et al., 2011). We used at most four reaction steps and a diameter of eight chemical bonds around the reaction center. Those conservative parameters were used to limit the strength of the substrate promiscuity hypothesis and to limit our results to pathways most likely to compete with known pathways.

### Strains, Genomic DNA, Plasmids, and Primers

Bacterial strains and plasmids used in this study are listed in Table 2. The *E. coli* strain DH5α was used as general cloning host, and *B. methanolicus* strains MGA3 and M168-20 were used as expression hosts. The following strains were the source of genetic material for cloning of the 5AVA synthesis pathways: *E. coli* MG1655, *Rhodococcus qingshengii* DSM45257, *Paenarthrobacter aurescens* DSM20116, *Kocuria rosea* DSM20447, *Peribacillus simplex* DSM1321, and *P. putida* KT2440. The L-lysine-α-oxidase-coding regions from *Trichoderma viride* (GenBank AB937978.1) and *S. japonicus* (GenBank AB970726.1) were codon-optimized for *B. methanolicus* MGA3 expression and synthesized by Twist Biosciences (Supplementary Table S1 and Supplementary Material). The *davBA* operons from alternative hosts *Williamsia sterculiae* CPCC 203464, *Roseobacter denitrificans* OCh 114 strain DSM 7001, and *Parageobacillus caldoxylosilyticus* B4119 (*davA* only) were codon-optimized for expression in *B. methanolicus*, synthesized and provided in the pUC57 plasmid from GenScript (Supplementary Table S1 and Supplementary Material). Isolated genomic DNA of *Bacillus megaterium* DSM32 was purchased from German Collection of Microorganisms and Cell Cultures GmbH (DSMZ). All primers (Sigma-Aldrich) used in this research are listed in Table 2.

**TABLE 2:** Bacterial strains, plasmids, and primers used in this study.

**Strain name**	**Relevant characteristics**	**References**
*Escherichia coli* DH5α	General cloning host, F-*thi*-1 *endA*1 *hsdR*17(r-,m-) *supE*44 _*lacU*169 (_80*lacZ*_M15) *recA*1 *gyrA*96 *relA*1	StrataGene
*E. coli* MG1655	Wild-type strain	ATCC 47076
*Bacillus methanolicus* MGA3	Wild-type strain	ATCC 53907
*Bacillus methanolicus* M160-20	1^st^-generation S-(2-aminoethyl) cysteine-resistant mutant of MGA3; L-lysine overproducer	Brautaset et al., 2010
*Rhodococcus qingshengii* DSM45257	Wild-type strain	DSM45257
*Paenarthrobacter aurescens* DSM20116	Wild-type strain	DSM20116
*Kocuria rosea* DSM20447	Wild-type strain	DSM20447
*Peribacillus simplex* DSM1321	Wild-type strain	DSM1321
*Pseudomonas putida* KT2440	Wild-type strain	DSM6125

**Genomic DNA**	**Relevant characteristics**	**References**

*Bacillus megaterium* DSM32	Wild-type strain	DSM32

**Plasmid**	**Relevant characteristics**	**References**

pBV2xp	Kan^*R*^; derivative of pHCMC04 for gene expression under control of the xylose-inducible promoter.	Drejer et al., 2020
pTH1mp	Cm^*R*^; derivative of pTH1mp-*lysC* for gene expression under control of the *mdh* promoter. The *lysC* gene was replaced with multiple cloning site.	Irla et al., 2016
pMI2mp	Cm^*R*^; Low copy number derivative (in *E. coli*) of pTH1mp	Drejer et al., 2020
pBV2xp-*davBA*^*Pp*^	Kan^*R*^; pBV2xp derivative for expression of the *P. putida davBA* operon under control of the xylose-inducible promoter.	This study
pBV2xp-*davBA*^*Ws*^	Kan^*R*^; pBV2xp derivative for expression of the *W. sterculiae davBA* operon under control of the inducible xylose-inducible ose promoter.	This study
pBV2xp-*davBA*^*Rd*^	Kan^*R*^; pBV2xp derivative for expression of the *R. denitrificans davBA* operon under control of the xylose-inducible promoter.	This study
pBV2xp-*davB*^*Ws*^-*davA*^*Pc*^	Kan^*R*^; pBV2xp derivative for expression of the synthetic operon containing *davB* from *W. sterculiae* and *davA* from *P. caldoxylosilyticus*. Expression under control of the xylose-inducible promoter.	This study
pBV2xp-*davA*^*Pc*^-*davB*^*Rd*^	Kan^*R*^; pBV2xp derivative for expression of the synthetic operon containing *davA* from *P. caldoxylosilyticus* and *davB* from *R. denitrificans*. Expression under control of the xylose-inducible promoter.	This study
pBV2xp-*davB*^*Pp*^	Kan^*R*^; pBV2xp derivative for expression of the *P. putida davB* gene under control of the xylose-inducible promoter.	This study
pBV2xp-*davB*^*Ws*^	Kan^*R*^; pBV2xp derivative for expression of the *W. sterculiae davB* gene under control of the xylose-inducible promoter.	This study
pMI2mp-*davA*^*Pc*^	Cm^*R*^; Derivative of pMI2mp for expression of *P. caldoxylosilyticus davA* gene under control of the constitutive *mdh* promoter.	This study
pMI2mp-*davA*^*Pp*^	Cm^*R*^; Derivative of pMI2mp for expression of *P. putida davA* gene under control of the constitutive *mdh* promoter.	This study
pBV2xp-*raiP*^*Ps*^	Kan^*R*^; pBV2xp-derived expression of *raiP* gene from *P. simplex*, under control of the xylose-inducible promoter	This study
pBV2xp-*raiP*^*Sj*^	Kan^*R*^; pBV2xp-derived expression of codon-optimized *raiP* gene from *S. japonicus*, under control of the xylose-inducible promoter	This study
pBV2xp-*raiP*^*Tv*^	Kan^*R*^; pBV2xp-derived expression of codon-optimized *raiP* gene from *T. viride*, under control of the xylose-inducible promoter	This study
pTH1mp-*cadA*	Cm^*R*^; Derivative of pTH1mp for expression of *E. coli* MG1655*-*derived *cadA* gene under control of the constitutive *mdh* promoter.	Nærdal et al., 2015
pTH1mp-*katA*	Cm^*R*^; Derivative of pTH1mp for expression of *B. methanolicus-*derived *katA* gene under control of the constitutive *mdh* promoter.	This study
pBV2xp-AVA^*Ec*^	Kan^*R*^; pBV2xp derivative for expression of the *E. coli* MG1655*-*derived genes *patDA* under control of the xylose-inducible promoter.	This study
pBV2xp-AVA^*Bm*^	Kan^*R*^; pBV2xp derivative for expression of the *B. megaterium* DSM32*-*derived genes *patDA* under control of the xylose-inducible promoter.	This study
pBV2xp-AVA^*Pp*^	Kan^*R*^; pBV2xp derivative for expression of *P. putida* KT2440*-*derived *spuI, spuC, kauB*, and *pauD2* genes under control of the xylose-inducible promoter.	This study
pBV2xp-AVA^*Rq*^	Kan^*R*^; pBV2xp derivative for expression of the *R. qingshengii* DSM45257-derived *puo* and *E. coli* MG1655*-*derived *patD* genes under control of the xylose-inducible promoter.	This study
pBV2xp-AVA^*Pa*^	Kan^*R*^; pBV2xp derivative for expression of the *P. aurescens* DSM20116-derived *puo* and *E. coli* MG1655*-*derived *patD* genes under control of the xylose-inducible promoter.	This study
pBV2xp-AVA^*Kr*^	Kan^*R*^; pBV2xp derivative for expression of the *K. rosea* DSM20447-derived *puo* and *E. coli* MG1655*-*derived *patD* genes under control of the xylose-inducible promoter.	This study

**Primer**	**Sequence 5′ → 3′**	**Characteristics**

davBA_Pp_F1	atagttgatggataaacttgttcacttaaggaggtagtacatatgaacaagaagaaccgcc	*davBA* from *P. putida;* fw
davBA_Pp_R1	aacgacggccagtgaattcgagctcactagttatcagcctttacgcaggtg	*davBA* from *P. putida;* rv
davB_Pp_F1	gatggataaacttgttcacttaagg	*davB* from *P. putida for* pBV2xp-*davB*^*Pp*^*;* fw
davB_Pp_R1	acggccagtgaattcgagctcaatccgccagggcgatc	*davB* from *P. putida for* pBV2xp-*davB*^*Pp*^*;* rv
davA_Pc_F1	ccagattagcatttaaactagttttgtaaacaattacataaataggaggtagtacatatggaaacatcatatgaaattgcac	*davA* from *P. caldoxylosilyticus for* pMI2mp-*davA*^*Pc*^*;* fw
davA_Pc_R1	tctagacctatggcgggtaccttaataaacatctgttcttctttcattcatc	*davA* from *P. caldoxylosilyticus for* pMI2mp-*davA*^*Pc*^*;* rv
davB_Ws_F1	ggataaacttgttcacttaaggaggtagtacatatgagagttacaacatcagttgg	*davB* from *W. sterculiae for* pBV2xp-*davB*^*Ws*^*;* fw
davB_Ws_R1	acggccagtgaattcgagctcttataatccaatatcaagtggtcc	*davB* from *W. sterculiae for* pBV2xp-*davB*^*Ws*^*;* rv
davA_Pp_F1	ccagattagcatttaaactagttttgtaaacaattacataaataggaggtagtacatatgcgcatcgctctgtacc	*dava* from *P. putida for* pMI2mp-*dava*^*Pp*^*;* fw
davA_Pp_R1	tctagacctatggcgggtacctcagcctttacgcaggtgc	*dava* from *P. putida for* pMI2mp-*dava*^*Pp*^*;* rv
raippsfw	cttgttcacttaagggggaaatggctatgctcgctgtgatcagaaatggccttgg	*raiP* from *P. simplex* fw
raippsrv	gccagtgaattcgagctcatggtacggatcttaaaaaggctcactcaatgttctaggc	*raiP* from *P. simplex* rv
raipsjfw	cttgttcacttaagggggaaatggctatggaacatttagcagattgtttagaag	*raiP* from *S. japonicus* fw
raipsjrv	gccagtgaattcgagctcatggtacggatcttataattcatcttttgtatgttcaattg	*raiP* from *S. japonicus* rv
raiptvfw	cttgttcacttaagggggaaatggctatggataatgttgattttgcagaatctg	*raiP* from *T. viride* fw
raiptvrv	gccagtgaattcgagctcatggtacggatcttaaattttaacttgatattcttttgg	*raiP* from *P. viride* rv
Katafw	gtaaacaattacataaataggaggtagtagtacatgaccacaaataagaaaaaacttactacaagc	*katA* from *B. methanolicus* fw
katarv	ggatccccgggaattcaagctttaaacatgttaaactttcttttgtacaggtaaacctagac	*katA* from *B. methanolicus* rv
AVA1	ttcacttaagggggaaatggcaaatggatcgtacagtcgttaaaa	*patDA* from *B. megaterium;* fw
AVA2	acgacggccagtgaattcgagctttattggtggttcagctcatt	*patDA* from *B. megaterium;* fw
AVA3	ttcacttaagggggaaatggcaaatgtcggtacccccgcgtgccgttcagcttaac	*spuI* from *P. putida*; fw
AVA4	ttacacggtatgcaggtaccag	*spuI* from *P. putida*; rv
AVA5	tggtacctgcataccgtgtaatacataaataggaggtagtaagaatgagcgtcaacaacccgcaaacccgtgaatg	*spuC* from *P. putida*; fw
AVA6	ttattgaatcgcctcaagggtcaggtccag	*spuC* from *P. putida*; rv
AVA7	acccttgaggcgattcaataatacataaataggaggtagtaagaatgaccaccctgacccgtgcggactgggaacaa	*kauB* from *P. putida*; fw
AVA8	ttacagcttgatccaggtcgccttcagctcgg	*kauB* from *P. putida*; rv
AVA9	cgacctggatcaagctgtaatacataaataggaggtagtaagaatgtcgttacgcatctgcatcc	*pauD2* from *P. putida*; fw
AVA10	acgacggccagtgaattcgagctttacgcggcgctgtcgccggcctttga	*pauD2* from *P. putida*; rv
AVA11	ttcacttaagggggaaatggcaaatgcaacataagttactgattaacggagaactggttag	*patD* from *E. coli*; fw
AVA12	ttaatgtttaaccatgacgtggcggacga	*patD* from *E. coli*; rv
AVA13	cacgtcatggttaaacattaatacataaataggaggtagtaagaatgaacaggttaccttcgagcgcatcggctttag	*patA* from *E. coli*; fw
AVA14	acgacggccagtgaattcgagctttacgcttcttcgacacttactcgcatgg	*patA* from *E. coli*; rv
AVA23	ttcacttaagggggaaatggcaaatgaacctaattcattttagtgtgaagg	*puo* from *Kocuria rosea*; fw
AVA29	tcttactacctcctatttatgtaattgtttactcatcgctccgcgcccgtca	*puo* from *Kocuria rosea*; rw
AVA25	ttcacttaagggggaaatggcaaatgcagaatcttgatcgcgacgttgtgatcgtcgg	*puo* from *P. aurescens*; fw
AVA30	tcttactacctcctatttatgtaattgtttactcaggcgacaggtacagaagccaacttgtt	*puo* from *P. aurescens*; rv
AVA27	ttcacttaagggggaaatggcaaatgcctactctccagagagacgttgcaatcgt	*puo* from *R. qingshengii*; fw
AVA31	tcttactacctcctatttatgtaattgtttactcaggccttgctgcgagcgatgatgt	*puo* from *R. qingshengii*; rv
AVA32	gtaaacaattacataaataggaggtagtaagaatgcaacataagttactgattaacggagaactggttag	*patD* from *E. coli* (for *puo*-*patD*); fw
AVA33	acgacggccagtgaattcgagctttaatgtttaaccatgacgtggcggacga	*patD* from *E. coli* (for *puo*-*patD*); rv
MI09	gataccaaatactgtccttctagtgtagccg	SDM of *ori* pUC9; fw
MI10	cggctacactagaaggacagtatttggtatc	SDM of *ori* pUC9; rv

*Cm^*R*^, chloramphenicol resistance; Kan^*R*^, kanamycin resistance.*

### Molecular Cloning

The *E. coli* DH5α competent cells were prepared according to the calcium chloride protocol as described in Green and Rogers (2013) or purchased as chemically competent NEB 5-α *E. coli* cells (New England Biolabs). All standard molecular cloning procedures were carried out as described in Sambrook and Russell (2001) or according to manuals provided by producers. Chromosomal DNA was isolated as described in Eikmanns et al. (1994). PCR products were amplified using CloneAmp HiFi PCR Premix (Takara) and purified using a QIAquick PCR Purification Kit from Qiagen. DNA fragments were separated using 8 g l^–1^ SeaKem LE Agarose gels (Lonza) and isolated using a QIAquick Gel Extraction Kit (Qiagen). The colony PCR was performed using GoTaq DNA Polymerase (Promega). The sequences of cloned DNA fragments were confirmed by Sanger sequencing (Eurofins). *B. methanolicus* MGA3 was made electrocompetent and transformed by electroporation as described previously (Jakobsen et al., 2006). Recombinant DNA was assembled *in vitro* by means of the isothermal DNA assembly method (Gibson et al., 2009), employing the NEBuilder HiFi DNA Assembly Kit or ligation with T4 DNA ligase. pMI2mp plasmid was obtained *via* site-directed mutagenesis (SDM) of pTH1mp performed as previously described with CloneAmp HiFi PCR Premix (Liu and Naismith, 2008). The detailed description of plasmid creation is presented in Supplementary Material.

### Media and Conditions for Shake Flask Cultivations

*E. coli* and *P. putida* strains were cultivated at 37°C in Lysogeny Broth (LB) or on LB agar plates supplemented with antibiotics when necessary. *P. aurescens* DSM2011 and *K. rosea* DSM20447 were cultivated at 30°C and 225 rpm in medium 53 (casein peptone, tryptic digest, 10.0 g l^–1^, yeast extract, 5.0 g l^–1^, glucose, 5.0 g l^–1^, NaCl, 5.0 g l^–1^; pH adjusted to 7.2–7.4); *R. qingshengii* DSM45257 was grown at 28°C and 225 rpm in medium 65 (glucose, 4.0 g l^–1^, yeast extract, 4.0 g l^–1^, malt extract, 10.0 g l^–1^; adjusted to pH to 7.2); and *P. simplex* DSM1321 was cultivated in nutrient medium (peptone 5 g l^–1^ and meat extract 3 g l^–1^; pH adjusted to 7.0) at 30°C and 200 rpm. For preparation of crude extracts, electrocompetent cells and transformation *B. methanolicus* strains were cultured at 50°C in SOB medium (Difco) supplemented with antibiotics when necessary. For 5AVA production experiments, recombinant *B. methanolicus* strains were cultivated in 250-ml baffled shake flasks at 50°C and 200 rpm in 40 or 50 ml MVcM medium containing 200 mM methanol. The MVcM medium contained the following, in 1 l of distilled water: K_2_HPO_4_, 4.09 g; NaH_2_PO_4_^∗^H_2_O, 1.49 g; (NH_4_)_2_SO_4_, 2.11 g; it was adjusted to pH 7.2 before autoclaving. The MVcM medium was supplemented with 1 ml 1 M MgSO_4_^∗^7H_2_O solution, 1 ml trace element solution, and 1 ml vitamin solution (Schendel et al., 1990). One mole of MgSO_4_^∗^7H_2_O solution contained 246.47 g of MgSO_4_^∗^7H_2_O in 1 l of distilled water. The trace element solution contained the following, in 1 l of distilled water: FeSO_4_^∗^7H_2_0, 5.56 g; CuSO_4_^∗^2H_2_O, 27.28 mg; CaCl_2_^∗^2H_2_O, 7.35 g; CoCl_2_^∗^6H_2_O, 40.50 mg; MnCl_2_^∗^4H_2_O, 9.90 g; ZnSO_4_^∗^7H_2_O, 287.54 mg; Na_2_MoO_4_^∗^2H_2_O, 48.40 mg; H_3_BO_3_, 30.92 mg; and HCl, 80 ml. The vitamin solution contained the following, in 1 l of distilled water: biotin, thiamine hydrochloride, riboflavin, D-calcium pantothenate, pyridoxine hydrochloride, nicotinamide, 0.1 g each; p-aminobenzoic acid, 0.02 g; folic acid, vitamin B_12_ and lipoic acid, 0.01 g each (Schendel et al., 1990). When needed, 10 g l^–1^ xylose (v/v) was added for induction. For precultures, a minimal medium supplemented with 0.25 g l^–1^ yeast extract, designated MVcMY, was used. Antibiotics (chloramphenicol, 5 μg ml^–1^ and/or kanamycin, 25 μg ml^–1^) were supplemented as necessary. Cultivations were performed in triplicates with start OD_600_ of 0.1–0.2. Growth was monitored by measuring OD_600_ with a cell density meter (WPA CO 8000 Biowave).

### Determination of Amino Acid Concentration

For the analysis of amino acid concentrations, 1 ml of the culture sample was taken from the bacterial cultures and centrifuged for 10 min at 11,000 rpm. Extracellular amino acids were quantified by means of high-pressure liquid chromatography (HPLC, Waters Alliance e2695 Separations Module). The samples underwent FMOC-Cl (fluorenylmethyloxycarbonyl chloride) derivatization before the analysis, according to the protocol described before (Haas et al., 2014), and were separated on a column (Symmetry C18 Column, 100 Å, 3.5 μm, 4.6 mm × 75 mm, Waters) according to the gradient flow presented in Table 3, where A is an elution buffer 50 mM Na-acetate pH = 4.2 and B is an organic solvent, acetonitrile. The detection was performed with a Waters 2475 HPLC Multi Fluorescence Detector (Waters), with excitation at 265 nm and emission at 315 nm.

**TABLE 3:** Determined parameters of mobile phase gradient conditions in a HPLC separation of FMOC-derivatized amino acids.

**Program time [min]**	**Flow rate [ml min^–1^]**	**%A**	**%B**
	1.3	62.0	38.0
5	1.3	62.0	38.0
12	1.3	43.0	57.0
14	1.3	24.0	76.0
15	1.3	43.0	57.0
18	1.3	620	38.0

*Mobile phase consists of elution buffer 50 mM Na-acetate pH = 4.2 (A) and organic solvent acetonitrile (B).*

### Enzyme Assays

In order to determine enzymatic activity, crude extracts of recombinant *B. methanolicus* cells were prepared according to Drejer et al. (2020). *B. methanolicus* strains were inoculated in SOB medium and grown to exponential phase (OD_600_ = 0.8). Recombinant expression was induced by addition of 10 g l^–1^ xylose 2 h after inoculation. A total amount of 50 ml culture broth was harvested by centrifugation at 7,500 rpm and 4°C for 15 min and washed twice in ice-cold buffer used for specific enzyme assay before storing at −80°C. The cells were thawed in ice and disrupted by sonication using a Fisherbrand Sonic Dismembrator (FB-505) with 40% amplitude with 2 s on and 1 s off-pulse cycles for 7 min. Cell debris was then removed by centrifugation (at 14, 000 rpm and 4°C for 1 h). Protein concentrations were determined by Bradford assay (Bradford, 1976), using bovine albumin serum (Sigma) as standard.

L-Lysine α-oxidase activity was assayed by measuring the rate of hydrogen peroxide formation, as described elsewhere (Tani et al., 2015a). The reaction was initiated by adding crude extracts from *B. methanolicus* strains to the reaction media (50°C) consisting of 100 mM L-lysine and 50 mM pH 7 phosphate buffer, resulting in a total volume of 1 ml. Next, the sample was quenched by addition of 50 μl 2 M HCl. After neutralization with 50 μl 2 M NaOH, 200 μl of the mixture was withdrawn and transferred to 800 μl of a second reaction mixture containing 50 mM pH 6 phosphate buffer, 30 mM phenol, 2 units ml^–1^ peroxidase from horseradish (Sigma) and 0.5 mM 4-aminoantipyrine. Formation of quinoneimine dye from oxidative coupling of phenol and 4-aminoantipyrine (Job et al., 2002) was determined by measuring absorbance at 505 nm using a Cary 100 Bio UV-visible spectrophotometer (Varian). One unit (U) of RaiP activity was defined as the amount of enzyme that catalyzes the formation of 1 μmol hydrogen peroxide per minute.

Catalytic activities of PatA and PatD or putrescine oxidase and PatD were measured by using a coupled reaction, and cadaverine was used as substrate instead of putrescine, as previously described elsewhere, with modifications (Jorge et al., 2017). The 1-ml assay mix contained 0.1 M Tris–HCl pH 8.0, 1.5 mM α-ketoglutarate, 2.5 mM cadaverine, 0.1 mM pyridoxal-5′-phosphate, and 0.3 mM NAD. In this coupled reaction, cadaverine was converted to 5AVA *via* 5-aminopentanal and one unit of coupled enzyme activity was defined as the amount of the enzyme that formed 1 μmol of NADH (ε340 nm = 6.22 mM^–1^ cm^–1^) per minute at 50°C.

The coupled DavAB assay was performed as described in Liu et al. (2014) with some modifications. Five hundred microliters of crude extract was added into 50-ml Falcon tubes filled with 4 ml 100 mM phosphate buffer pH 7.0 supplemented with 10 g l^–1^ L-lysine. The tubes were incubated for 40 h at 30 or 50°C with stirring at 200 rpm. The samples for quantification of 5AVA concentration through HPLC (see section “Determination of Amino Acid Concentration”) were taken at the beginning of incubation, after 16 h and after 40 h.

## Results and Discussion

### Selection, Design, and Construction of Heterologous Biosynthetic Pathways for 5AVA Biosynthesis in *B. methanolicus*

Due to the fact that *B. methanolicus* is a thermophile, a typical issue concerning implementation of biosynthetic pathways from heterologous hosts is the lack of thermostability of the transferred enzymes. It was shown before that a screening of diverse donor organisms allows to identify pathways active at 50°C and leads to increased product titers (Irla et al., 2017; Drejer et al., 2020). In order to extend the scope of our screening, we have constructed 26 strains with five different 5AVA biosynthetic pathways, which are presented in Figure 1, derived from diverse donors. Two pathways that directly convert L-lysine to 5AVA were chosen: the DavBA pathway (Figure 1A) and the RaiP pathway (Figure 1B), as well as three pathways that use cadaverine as an intermediate: the SpuI pathway (Figure 1C), the PatA pathway (Figure 1D), and the Puo pathway (Figure 1E).

The genes encoding the core part of those pathways are cloned into a θ-replication, low copy number derivative of pHCMC04 plasmid, pBV2xp, under control of a *B. megaterium*-derived, xylose-inducible promoter, and the genes encoding any ancillary enzymes are cloned into pTH1mp or pMI2mp plasmids, which are compatible to pBV2xp, under control of the *mdh* promoter (Irla et al., 2016). The plasmids with genes encoding desired pathways were constructed as described fully in the Supplementary Material and then used to transform *B. methanolicus* cells leading to formation of strains presented in Table 4.

**TABLE 4:** List of *B. methanolicus* strains used in this study with abbreviated strain names.

**Abbreviated strain name**	**Recombinant *B. methanolicus* strains created in this study**
MGA3_EV	MGA3(pBV2xp)
MGA3_DavBA^*Pp*^	MGA3(pBV2xp-*davBA*^*Pp*^)
MGA3_DavBA^*Ws*^	MGA3(pBV2xp-*davBA*^*Ws*^)
MGA3_DavBA^*Rd*^	MGA3(pBV2xp-*davBA*^*Rd*^)
MGA3_DavB^*Ws*^A^*Pc*^	MGA3(pBV2xp-*davB*^*Ws*^-*davA*^*Pc*^)
MGA3_DavA^*Pc*^B^*Rd*^	MGA3(pBV2xp-*davA*^*Pc*^-*davB*^*Rd*^)
MGA3_DavB^*Pp*^A^*Pc*^(2p)	MGA3(pMI2mp-*davA*^*Pc*^)(pBV2xp-*davB*^*Pp*^)
MGA3_DavB^*Ws*^A^*Pc*^(2p)	MGA3(pMI2mp-*davA*^*Pc*^)(pBV2xp-*davB*^*Ws*^)
M168-20_EV	M168-20(pBV2xp)
M168-20_DavBA^*Pp*^	M168-20(pBV2xp-*davBA*^*Pp*^)
M168-20_DavA^*Pp*^B^*Pp*^(2p)	M168-20(pMI2mp-*davA*^*Pp*^)(pBV2xp-*davB*^*Pp*^)
M168-20_DavA^*Pp*^B^*Ws*^(2p)	M168-20(pMI2mp-*davA*^*Pp*^)(pBV2xp-*davB*^*Ws*^)
MGA3_RaiP^*Ps*^	MGA3(pBV2xp-*raiP*^*Ps*^)
MGA3_RaiP^*Sj*^	MGA3(pBV2xp-*raiP*^*Sj*^)
MGA3_RaiP^*Tv*^	MGA3(pBV2xp-*raiP*^*Tv*^)
M168-20_RaiP^*Ps*^	M168-20 (pBV2xp-*raiP*^*Ps*^)
M168-20_RaiP^*Sj*^	M168-20(pBV2xp-*raiP*^*Sj*^)
M168-20_RaiP^*Tv*^	M168-20 (pBV2xp-*raiP*^*Tv*^)
MGA3_Cad	MGA3(pTH1mp-*cadA*)(pBV2xp)
MGA3_PatA^*Ec*^	MGA3(pTH1mp-*cadA*)(pBV2xp-AVA^*Ec*^)
MGA3_PatA^*Bm*^	MGA3(pTH1mp-*cadA*)(pBV2xp-AVA^*Bm*^)
MGA3_SpuI	MGA3(pTH1mp-*cadA*)(pBV2xp-AVA^*Pp*^)
MGA3_Kat	MGA3(pTH1mp-*katA*)(pBV2xp)
MGA3_Puo^*Kr*^	MGA3(pTH1mp-*katA*)(pBV2xp-AVA^*Kr*^)
MGA3_Puo^*Pa*^	MGA3(pTH1mp-*katA*)(pBV2xp AVA^*Pa*^)
MGA3_Puo^*Rq*^	MGA3(pTH1mp-*katA*)(pBV2xp-AVA^*Rq*^)

With help of retrosynthesis analysis, we have considered two pathways that utilize L-lysine directly as precursor and that utilize either DavB (EC 1.13.12.2) and DavA (EC 3.5.1.30) activity (DavBA pathway, Figure 1A) or RaiP (EC 1.4.3.14) in the presence of H_2_O_2_ (RaiP pathway, Figure 1B) for further conversion into 5AVA. For DavBA production, three different *davBA* operons from the following mesophilic organisms were applied: *P. putida*, *W. sterculiae*, and *R. denitrificans.* We could not identify a complete *davBA* operon from a thermophilic host; however, thermophilic *P. caldoxylosilyticus* possesses a putative *davA* gene and was also included in this study. All selected *davBA* operons were codon-optimized and cloned into the pBV2xp vector under control of the xylose-inducible promoter as described in the Supplementary Material. The finished vectors were used to create the following *B. methanolicus* strains: MGA3_DavBA^*Pp*^, MGA3_DavBA^*Ws*^, MGA3_DavBA^*Rd*^, MGA3_DavB^*Ws*^A^*Pc*^, and MGA3_DavA^*Pc*^B^*Rd*^ (Table 4). Furthermore, selected *davBA* operons were expressed as single genes using compatible pBV2xp and pMI2mp plasmids for gene expression (Supplementary Material). The *davB* genes from *P. putida* and *W. sterculiae* were cloned under control of the xylose-inducible promoter in plasmid pBV2xp, while the *davA* gene from *P. caldoxylosilyticus* was cloned into the pMI2mp plasmid under control of the *mdh* promoter constitutively active in methylotrophic conditions. The combination of two plasmids (2p) expressing single genes resulted in creation of the following *B. methanolicus* strains: MGA3_DavB^*Pp*^A^*Pc*^(2p) and MGA3_DavB^*Ws*^A^*Pc*^(2p) (Table 4).

For expression of the RaiP pathway, the *B. methanolicus* strains MGA3_RaiP^*Ps*^, MGA3_RaiP^*Sj*^, and MGA3_RaiP^*Tv*^ (Table 4) carried heterologous *raiP* gene sequences from the prokaryote *P. simplex* and from the eukaryotic genetic donors *S. japonicus* and *T. viride*, respectively, the two latter with characterized RaiP activity (Arinbasarova et al., 2012; Tani et al., 2015a). The full length of codon-optimized sequences derived from *S. japonicus* and *T. viride* is present in the Supplementary Table S1. The original *S. japonicus* sequence encodes a protein with 617 amino acids and has a 52.2% GC content, while the sequence codon optimized for *B. methanolicus* has a GC content of 29%. The *T. viride*-derivative sequence was adjusted from the GC content of 42.5 to 28.6%. The substitution of nucleotides did not alter their coding amino acid sequences.

Among the pathways using cadaverine formed from L-lysine through activity of *E. coli*-derived lysine decarboxylase CadA (EC 4.1.1.18, encoded by *cadA*) as an intermediate, we considered a multistep diamine catabolic pathway of *P. aeruginosa* PAOI (SpuI pathway, Figure 1C) (Yao et al., 2011). In order to test this pathway for methanol-based 5AVA production, the MGA3_SpuI strain was constructed through transformation of *B. methanolicus* wild type with two vectors pTH1mp-*cadA* and pBV2xp-AVA^*Pp*^, the first one carrying the *cadA* gene and the latter the genes encoding the SpuI pathway (Table 4 and Supplementary Material). The SpuI pathway that converts cadaverine to 5AVA is composed of the following enzymes: glutamylpolyamine synthetase (EC 6.3.1.2, SpuI), polyamine:pyruvate transaminase (EC 2.6.1.113, SpuC), aldehyde dehydrogenase (EC 1.2.1.3, KauB), and glutamine amidotransferase class I (EC 6.3.5.2, PauD2) (Yao et al., 2011).

Another pathway, also predicted by our retrosynthesis analysis, potentially leading to production of 5AVA from L-lysine is a three-step pathway composed of CadA, PatA (EC 2.6.1.82, PatA), and 5-aminopentanal dehydrogenase (EC 1.2.1.19, PatD) (PatA pathway, Figure 1D). In order to test this pathway, two strains were constructed, MGA3_PatA^*Ec*^ and MGA3_PatA^*Bm*^, through transformation *of B. methanolicus* with pTH1mp-*cadA* plasmid, and pBV2xp-AVA^*Ec*^ or pBV2xp-AVA^*Bm*^, respectively (Table 4). As described in the Supplementary Material, the lysine decarboxylase-encoding gene (*cadA*) was placed under control of the *mdh* promoter in a rolling circle vector pTH1mp. The *E. coli*-derived *patAD* operon encoding previously characterized enzymes was placed under control of the xylose-inducible promoter in pBV2xp, resulting in pBV2xp-AVA^*Ec*^ (Samsonova et al., 2003). The genes of the *patAD* operon in *B. megaterium* were identified based on a BLAST search of its genome and were cloned into pBV2xp, yielding pBV2xp-AVA^*Bm*^ (Altschul et al., 1990). While the existence of prior art makes it a solid candidate, we knew that its second step catalyzed by PatA may suffer from an unfavorable thermodynamic (predicted close to 0 kJ mol^–1^) (Noor et al., 2012).

In our study, we have also included a pathway confirmed through retrosynthesis analysis where the step of cadaverine transamination (PatA pathway, Figure 1D) is replaced by its oxidative deamination (Puo pathway, Figure 1E) because this reaction displays a more favorable thermodynamic (predicted close to −100 kl mol^–1^ in cell conditions) in comparison to PatA. While a cadaverine oxidase has not been identified before, it was shown that putrescine oxidase encoded by *puo* retains up to 14% of its maximal activity when cadaverine is used as a substrate (Okada et al., 1979; Ishizuka et al., 1993; van Hellemond et al., 2008; Lee and Kim, 2013). We have therefore decided to express three different versions of the *puo* gene derived from *K. rosea*, *P. aurescens*, and *R. qingshengii*, together with the *E. coli*-derived *patD* gene from the pBV2xp plasmid (for details see Supplementary Material), which led to creation of the following strains: MGA3_Puo^*Kr*^, MGA3_Puo^*Pa*^, and MGA3_Puo^*Rq*^, respectively (Table 4). In order to prevent oxidative stress caused by H_2_O_2_ formation, a native gene encoding catalase was homologously expressed from pTH1mp plasmid in all constructed strains.

### Testing Recombinant *B. methanolicus* Strains for 5AVA Production From Methanol

The plasmids designed and built as described in the above Section were used for transformation of wild-type *B. methanolicus* cells and resulted in the creation of 26 different strains (Table 4) which were then tested for their ability to synthetize 5AVA. All strains were cultivated in minimal medium supplemented with methanol as the sole carbon and energy source, and the 5AVA titer was evaluated after the strains had reached the stationary growth phase as described in the following sections.

#### Expression of the DavAB-Encoding Genes Resulted in no 5AVA Biosynthesis in *B. methanolicus*

In the first attempt, we heterologously expressed genes encoding the DavBA pathway in *B. methanolicus* MGA3 (Figure 1A). In addition to the well-known *davBA* operon from *P. putida* (gamma-proteobacteria), the alternative *davBA* operon from *W. sterculiae* (actinobacteria) and *davAB* from *R. denitrificans* (alpha-proteobacteria) were tested for 5AVA formation in *B. methanolicus* MGA3. Moreover, the only enzyme identified from a thermophilic host, DavA from *P. caldoxylosilyticus* (bacilli), was combined with the before mentioned lysine 2-monooxygenases (DavB). *P. caldoxylosilyticus* has a reported optimum growth temperature from 50 to 65°C (Fortina et al., 2001).

Several considerations were made with regard to strain design, namely, adjusting the GC content and the types of codons present in the open reading frames in the genomic DNA of a donor and designing suitable expression cassettes. In total, seven different *B. methanolicus* strains were constructed: MGA3(pBV2xp-*davBA*^*Pp*^) named MGA3_DavBA^*Pp*^, MGA3(pBV2xp-*davBA*^*Ws*^) named MGA3_DavBA^*Ws*^, MGA3(pBV2xp-*davBA*^*Rd*^) named MGA3_DavBA^*Rd*^, MGA3(pBV2xp-*davB*^*Ws*^-*davA*^*Pc*^) named MGA3_DavB^*Ws*^A^*Pc*^, MGA3(pBV2xp-*davA*^*Pc*^-*davB*^*Rd*^) named MGA3_DavA^*Pc*^B^*Rd*^, MGA3(pMI2mp-*davA*^*Pc*^)(pBV2xp-*davB*^*Pp*^) named MGA3_DavB^*Pp*^A^*Pc*^(2p), MGA3(pMI2mp-*davA*^*Pc*^) (pBV2xp-*davB*^*Ws*^) named MGA3_DavB^*Ws*^A^*Pc*^(2p) (Table 4). However, in none of the tested strains (MGA3_DavBA^*Pp*^, MGA3_DavBA^*Ws*^, MGA3_DavBA^*Rd*^, MGA3_DavB^*Ws*^A^*Pc*^, MGA3_DavA^*Pc*^B^*Rd*^), the active pathway was expressed; and followingly no 5AVA accumulation was observed during shake flask cultivations in any constructed strain (data not shown). The first reaction step from L-lysine to 5-aminopentanamide requires O_2_ (Figure 1A), and due to the high O_2_ demand to facilitate the assimilation of methanol, we also tested 5AVA formation from the alternative carbon source mannitol. Neither was this strategy successful. Furthermore, the DavAB pathway was also tested in the genetic background of L-lysine-overproducing *B. methanolicus* strain M160-20. Specifically, the following strains were constructed: M168-20_DavBA^*Pp*^, M168-20_DavA^*Pp*^B^*Pp*^(2p), and M168-20_DavA^*Pp*^B^*Ws*^(2p); however, none of them produced any detectable 5AVA (data not shown). Taken together, the DavBA pathway did not enable 5AVA formation. It is not clear whether this was caused by low enzymatic stability at 50°C (only *P. caldoxylosilyticus* is known to be thermophilic among the organisms found to be source organisms for the two genes). In order to exclude the effect of elevated temperature on the DavAB activity, we tested enzymatic activity at 30°C for selected strains (MGA3_DavBA^*Pp*^, MGA3_DavBA^*Ws*^, MGA3_DavBA^*Rd*^, MGA3_DavB^*Ws*^A^*Pc*^, and MGA3_DavA^*Pc*^B^*Rd*^); however, no DavAB activity was detected (data not shown). The reason why the functional DavAB pathway was not expressed in *B. methanolicus* remains unknown.

#### RaiP Pathway Is Functional in *B. methanolicus* and Supports 5AVA Production

**FIGURE 2:**
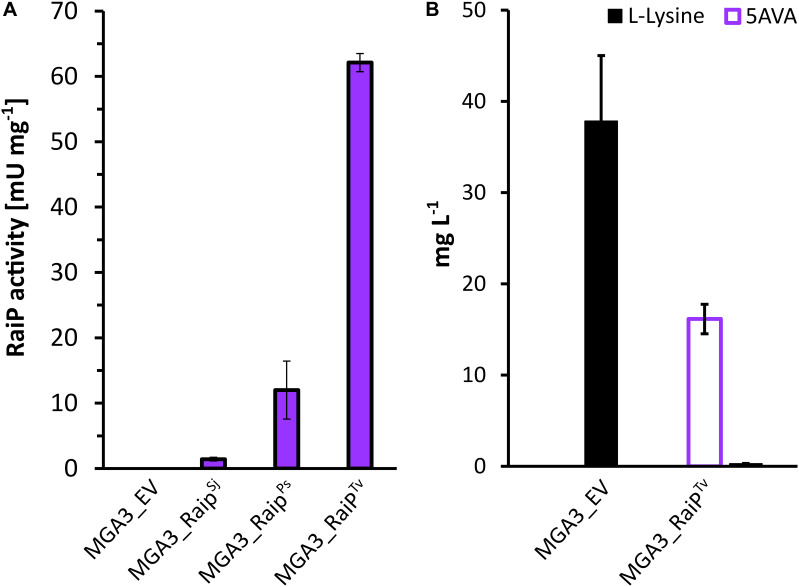
Evaluation of RaiP enzyme activity **(A)** and amino acids production **(B)** in recombinant *B. methanolicus* strains. *B. methanolicus* strains MGA3_EV, MGA3_RaiP^*Sj*^, MGA3_RaiP^*Ps*^, or MGA3_RaiP^*Tv*^ were cultivated in a shaking flask culture. The grown cells were harvested, washed twice with 50 mM phosphate buffer (pH 7.0), and disrupted by sonication. After centrifugation, the crude extracts were directly used for the RaiP assay. MGA3_EV and MGA3_RaiP^*Tv*^ were cultivated for 27 h, and supernatants were obtained by centrifugation for HPLC analysis. The error bars represent standard deviation of technical triplicates.

Methanol-based 5AVA biosynthesis was attempted *via* heterologous expression of RaiP encoding gene *raiP* in MGA3. The strains MGA3(pBV2xp-*raiP*^*Ps*^) named MGA3_RaiP^*Ps*^, MGA3(pBV2xp-*raiP*^*Sj*^) named MGA3_RaiP^*Sj*^, and MGA3(pBV2xp-*raiP*^*Tv*^) named MGA3_RaiP^*Tv*^ (Table 4) carry the *raiP* gene from the bacterium *P. simplex* and *raiP* genes with codon-optimized sequences from the eukaryotic donors *S. japonicus* and *T. viride*, respectively. The *T. viride*-derived RaiP was shown to be stable at temperatures up to 50°C (Arinbasarova et al., 2012). It is reported that the RaiP protein from *S. japonicus* is thermally stable for at least 1 h in temperatures up to 60°C, with its highest activity registered at 70°C (Tani et al., 2015b). Moreover, although there is no kinetic characterization of RaiP from *P. simplex* available, this bacterium is classified as mesophilic, with growth optimum at 30°C (Yumoto et al., 2004). To examine the activity of RaiP in the constructed *B. methanolicus* strains, L-lysine α-oxidase activity was measured at 50°C. While the empty vector control strain has shown no RaiP activity, the highest RaiP specific activity was observed in crude extracts from strain MGA3_RaiP^*Tv*^, being 62.1 ± 1.4 mU mg^–1^ (Figure 2A). The values of RaiP activity for strains MGA3_RaiP^*Ps*^ and MGA3_RaiP^*Sj*^ were 1.4 ± 0.3 mU mg^–1^ and 12.0 ± 4.4 mU mg^–1^, respectively (Figure 2A). It is not clear if the poor activity of heterologous RaiP from genetic donors *S. japonicus* and *P. simplex* was caused by low enzymatic stability at 50°C, and the reason for that remains to be investigated.

HPLC analysis of supernatant from MGA3_RaiP^*Tv*^ strain cultivated in minimal medium revealed 16.15 ± 1.62 mg L^–1^ 5AVA and 0.27 ± 0.04 mg L^–1^ L-lysine. In contrast, the L-lysine level in the MGA3 strain harboring the empty vector plasmid pBV2xp (MGA3_EV) was 37.8 ± 7.2 mg L^–1^ (Figure 2B). Even though a slight RaiP activity was observed in crude extract of the strains MGA3_RaiP^*Ps*^ and MGA3_RaiP^*Sj*^, no 5AVA production was observed for those strains (data not shown). Let us note here that the 5AVA titer in the methanol-based shaking flask fermentation of strain MGA3_RaiP^*Tv*^ was significantly inferior to that in previously reported glucose-based fermentations in *E. coli* (Cheng et al., 2018).

The value of the Michaelis–Menten constant for *T. viride*-derived RaiP for L-lysine has been estimated (*K*_m_ = 5.85 mg L^–1^) (Kusakabe et al., 1980). Therefore, the precursor levels in the *B. methanolicus* strains should not be a limiting factor for production of 5AVA. The RaiP-mediated production is mainly utilized in the L-lysine bioconversion approach, utilizing *E. coli* strains as whole-cell biocatalysts (Cheng et al., 2018, 2020, 2021) where high concentrations of the precursor were used; for example, the molar yield of 0.942 was obtained from 120 g l^–1^ L-lysine (Park et al., 2014). However, construction and testing of the *B. methanolicus* strains M168-20_RaiP^*Sj*^, M168-20_RaiP^*Ps*^, and M168-20_RaiP^*Tv*^ (Table 4), based on the L-lysine-over producing mutant M168-20 (Brautaset et al., 2010), did not result in any improved 5AVA production (data not shown).

The lack of 5AVA production in MGA3_RaiP^*Ps*^ and MGA3_RaiP^*Sj*^, as well as low 5AVA titer produced by strain MGA3_RaiP^*Tv*^, might be related to the spontaneous conversion step that follows RaiP activity. This could be a limiting factor for the RaiP-mediated production of 5AVA. Three compounds are produced in a reaction catalyzed by RaiP: α-ketolysine, NH_3_, and H_2_O_2_ (Mai-Prochnow et al., 2008; Cheng et al., 2018). In a second spontaneous step of 5AVA synthesis, the intermediate α-ketolysine is oxidatively decarboxylated to form 5AVA in the presence of H_2_O_2_ as an oxidizing agent. It was shown that the addition of H_2_O_2_ into the culture broth has led to an 18−fold increase of 5AVA titers in comparison with the control condition without H_2_O_2_ (final titer 29.12 g l^–1^) in a 5–l fermenter (Cheng et al., 2018). The RaiP-mediated 5AVA production may be increased by enzymatic conversion of α-ketolysine in an approach different to ours, where spontaneous reaction of oxidative decarboxylation occurs. Recently, an artificial synthetic pathway for the biosynthesis of 5AVA in *E. coli* was developed, consisting of three steps: conversion of L-lysine to α-ketolysine *via* RaiP, decarboxylation of α-ketolysine to produce 5-aminopentanal *via* α-ketoacid decarboxylase, and oxidation of 5-aminopentanal to 5AVA *via* aldehyde dehydrogenase. The expression of the artificial pathway resulted in a yield increase of 774% compared to the single gene pathway (Cheng et al., 2021). This approach is potentially a feasible strategy we have shown in our study that *E. coli*-derived PatD is active as a 5-aminopentanal dehydrogenase in *B. methanolicus* and participates in 5AVA biosynthesis (see Section “The PatA Pathway Supports 5AVA Accumulation in *B. methanolicus*).

#### Use of the SpuI Pathway Does Not Lead to 5AVA Production in *B. methanolicus*

Three different pathways that use cadaverine as an intermediate product have been tested for their feasibility for production of 5AVA in *B. methanolicus*. Cadaverine biosynthesis in *B. methanolicus* cells was enabled through the activity of lysine decarboxylase encoded by a heterologously expressed *cadA* (Nærdal et al., 2015). Cadaverine can be converted to 5AVA through activity of a multistep diamine catabolic pathway derived from *P. aeruginosa* PAOI (SpuI pathway, Figure 1C) (Yao et al., 2011). The MGA3(pTH1mp-*cadA*)(pBV2xp-AVA^*Pp*^) strain called MGA3_SpuI (Table 4) did not accumulate any 5AVA during methanol-based growth in minimal medium, despite the accumulation of the precursor, cadaverine, at the level of 118.8 ± 5.1 mg l^–1^ similar to the empty vector control strain (130.0 ± 5.3 mg l^–1^) (Table 5). The cadaverine titers of 130.0 ± 5.3 mg l^–1^ achieved by MGA3_Cad are higher than the L-lysine titer of 37.8 ± 7.2 mg L^–1^ achieved by MGA3_EV in this study (Figure 2B). This is in accordance with previous findings of Nærdal et al. (2011, 2015) who attributed high cadaverine titers for production strain in relation to L-lysine titer in empty vector control strain to a metabolic pull which deregulated flux through the L-lysine biosynthesis pathway.

**TABLE 5:** Growth rates, enzyme activities and L-lysine, cadaverine, and 5AVA final titers accumulated in growth media of recombinant MGA3 strains.

**Strain**	**Growth rate [h^–1^]**	**Coupled activity of PatAD or Puo-PatD [mU mg^–1^]**	**Lysine [mg l^–1^]**	**Cadaverine [mg l^–1^]**	**5AVA [mg l^–1^]**
MGA3_Cad	0.37 ± 0.01	0 ± 0	Not detected	123.0 ± 5.3	0.0 ± 0.0
MGA3_SpuI	0.33 ± 0.01	N.A.	Not detected	118.82 ± 5.1	0.0 ± 0.0
MGA3_PatA^*Ec*^	0.12 ± 0.02	7 ± 4	Not detected	1.47 ± 0.17	23.7 ± 2.7
MGA3_PatA^*Bm*^	0.15 ± 0.03	170 ± 37	Not detected	0.71 ± 0.11	8.3 ± 4.1
MGA3_Cad	0.35 ± 0.01	0 ± 0	Not detected	Supplemented (500 mg L^–1^)	0.0 ± 0.0
MGA3_SpuI	0.32 ± 0.00	N.A.	Not detected	Supplemented (500 mg L^–1^)	0.0 ± 0.0
MGA3_PatA^*Ec*^	0.14 ± 0.02	7 ± 4	Not detected	Supplemented (500 mg L^–1^)	31.8 ± 2.3
MGA3_PatA^*Bm*^	0.17 ± 0.04	170 ± 37	Not detected	Supplemented (500 mg L^–1^)	77.7 ± 5.5
MGA3_Kat	0.32 ± 0.00	0 ± 0	3.1 ± 0.5	Supplemented (500 mg L^–1^)	0.0 ± 0.0
MGA3_Puo^*Ec*^	0.28 ± 0.01	0 ± 0	5.0 ± 0.7	Supplemented (500 mg L^–1^)	0.0 ± 0.0
MGA3_Puo^*Pa*^	0.29 ± 0.01	0 ± 0	4.9 ± 0.9	Supplemented (500 mg L^–1^)	0.0 ± 0.0
MGA3_Puo^*Rq*^	0.29 ± 0.00	0 ± 0	3.7 ± 0.2	Supplemented (500 mg L^–1^)	0.0 ± 0.0

*The *B. methanolicus* strains expressing pathways that use cadaverine as an intermediate (SpuI, PatA, or Puo pathways) were cultivated for 24 h, and supernatants were obtained by centrifugation for HPLC analysis. Catalytic activities of PatA and PatD or Puo and PatD were measured by using a coupled reaction, and cadaverine was used as substrate (see Section “Enzyme Assays”). The standard deviation of technical triplicates is shown. NB: RaiP activity and 5AVA production for the RaiP pathway is shown Figure 2.*

#### The PatA Pathway Supports 5AVA Accumulation in *B. methanolicus*

In the next step, two versions of the PatA pathway (Figure 1D) derived from either *E. coli* or *B. megaterium* were tested in strains MGA3(pTH1mp-*cadA*)(pBV2xp-AVA^*Ec*^) named MGA3_PatA^*Ec*^ and MGA3(pTH1mp-*cadA*)(pBV2xp-AVA^*Bm*^) named MGA3 _PatA^*Bm*^ (Table 4), respectively. The optimal temperature of PatA derived from *E. coli* is 60°C, which means that it is a thermostable enzyme that should be active at 50°C, which is a temperature used for the production experiment. PatA was shown to have a broad substrate range including cadaverine and, in lower extent, spermidine, but not ornithine (Samsonova et al., 2003). This property was used by Jorge et al. (2017) who have shown in their study that it is possible to use PatA and PatD derived from *E. coli* to establish conversion of cadaverine to 5AVA, confirming experimentally the broad substrate range of those two enzymes. The *B. megaterium*-derived PatA was characterized only superficially with regard to its substrate spectrum and not optimal temperature or thermostability (Slabu et al., 2016); however, its host organism is known to have a wide temperature range for growth up to 45°C (Vary et al., 2007). The multiple-sequence alignment with *E. coli*-derived enzymes showed identity of 63 and 38% for PatA and PatD, respectively (Okada et al., 1979). Both *E. coli* and *B. megaterium*-derived versions of the pathway are functional in *B. methanolicus*, with the combined PatAD activity of 7 ± 4 mU and 170 ± 37 mU mg^–1^ (Table 5). Final 5AVA titers of 23.7 ± 2.7 and 8.3 ± 4.1 mg L^–1^ (Table 5) were achieved, which is considerably lower than 5AVA titers of 0.9 g l^–1^ obtained by wild-type *C. glutamicum* strain transformed with plasmids for expression of *ldcC* (coding for lysine decarboxylase) and *patDA* (Jorge et al., 2017). For both producer strains, the concentration of unconverted cadaverine is similar: 1.7 ± 0.1 mg l^–1^ and 1.5 ± 0.2 mg l^–1^ for MGA3_PatA^*Ec*^ and MGA3_PatA^*Bm*^, respectively (Table 5). While *K*_m_ for cadaverine has not been assessed, it has been shown to be 811 mg l^–1^ for putrescine for *E. coli*-derived PatA; assuming similar *K*_m_ for cadaverine, it may explain why full conversion of cadaverine has not occurred (Samsonova et al., 2003). Due to relatively high *K*_m_ for putrescine of PatA, we decided to test how supplementation with external cadaverine affects 5AVA accumulation. In fact, for both MGA3_PatA^*Ec*^ and MGA3_PatA^*Bm*^, 5AVA titers increased to 31.8 ± 2.3 and 77.7 ± 5.5, respectively, when the growth medium was supplemented with 500 mg l^–1^ cadaverine (Table 5). These results indicate that the enhancement of precursor supply is one potential target for subsequent metabolic engineering efforts to increase 5AVA titers. Another important consideration for activity of transaminase is availability of keto acid that acts as amino group acceptor. It was shown that *E. coli* and *B. megaterium*-derived PatA can use either pyruvate or 2-oxoglutarate as amino group acceptors (Slabu et al., 2016); the intracellular concentrations of those compounds in *B. methanolicus* MGA3 cells are 3.2 and 2.7 mM, respectively (Brautaset et al., 2003). Knowing that *K*_m_ for 2-oxoglutarate for *E. coli*-derived PatA is 19.0 mM (Samsonova et al., 2003), recovery of the keto acids may be a limitation for 5AVA accumulation. This issue could be potentially solved by heterologous production of alanine dehydrogenase or L-glutamate oxidase which catalyzes reactions where pyruvate or 2-oxoglutarate is produced (Böhmer et al., 1989; Sakamoto et al., 1990; Slabu et al., 2016).

#### Use of the Puo Pathway Leads to 5AVA Production in *B. methanolicus*

Lastly, a pathway that relies on an activity of the monooxygenase putrescine oxidase (Puo, EC 1.4.3.10) was tested (Figure 1E). Puo catalyzes the oxidative deamination of cadaverine *in lieu* of cadaverine transamination catalyzed by PatA. It was shown that different putrescine oxidases can use cadaverine as their substrate with 9–14% of their maximal activity shown when putrescine is a substrate (Desa, 1972; Okada et al., 1979; van Hellemond et al., 2008; Lee et al., 2013). Moreover, putrescine oxidases derived from *K. rosea* (*Micrococcus rubens*) and *Rhodococcus* are thermostable and optimal activity of *P. aurescens-*derived Puo is at 50°C (Desa, 1972; van Hellemond et al., 2008; Lee et al., 2013). The disadvantage of this pathway is that it requires O_2_, the supply of which may be difficult to control. Furthermore, due to formation of hydrogen peroxide in the reaction catalyzed by Puo, the oxidative stress may increase when this pathway is active. In order to avoid detrimental effect of hydrogen peroxide accumulation, catalase was overproduced in the recombinant strains containing the Puo pathway: MGA3(pTH1mp-*katA*)(pBV2xp-AVA^*Kr*^) named MGA3_Puo^*Kr*^, MGA3(pTH1mp-*katA*)(pBV2xp AVA^*Pa*^) named MGA3_Puo^*Pa*^, and MGA3(pTH1mp-*katA*)(pBV2xp-AVA^*Rq*^) named MGA3_Puo^*Rq*^ (Table 4). To achieve sufficient levels of the pathway precursor, cadaverine, we have decided not to rely on plasmid-based production of lysine decarboxylase and to add cadaverine to the growth medium, instead. The tested recombinant strains with the Puo pathway did not produce 5AVA, which is consistent with no Puo-PatD activity detected in crude extracts (Table 5). The Puo pathway was shown to be active in *C. glutamicum* where titer of 0.1 ± 0.0–0.4 ± 0.0 g l^–1^ 5AVA was achieved (Haupka et al., 2020).

## Conclusion

In the search for 5AVA production from the sustainable feedstock methanol, we have screened five pathways toward 5AVA biosynthesis in *B. methanolicus*. No 5AVA production was observed for DavBA, Puo, and SpuI pathways. However, the pathways relying on RaiP and PatA activities were functional in shake flask cultures of *B. methanolicus*, which led to 5AVA production from methanol for the first time, respectively, up to 16.15 ± 1.62 mg l^–1^ or 23.7 ± 2.7. RaiP and PatA pathways are targets for further optimizations which could increase the 5AVA titers in the constructed strains. For instance, the improvement of substrate utilization and H_2_O_2_ availability or decomposition efficiency might contribute to the increase in the yield of 5AVA. Moreover, our study shows that the availability of supplemented cadaverine has high impact on 5AVA titer when the PatA pathway is employed. Another factor that needs to be considered is tolerance to 5AVA, which was shown to be low (Haupka et al., 2021). Recently, adaptative laboratory evolution experiments resulted in the selection of a mutant strain of *B. methanolicus* that displays tolerance to approximately 46 g l^–1^ 5AVA (Haupka et al., 2021), which could be employed as a platform to develop high-titer 5AVA production strains. This shows that methanol has the potential to become a sustainable feedstock for the production of 5AVA.

## Data Availability Statement

The original contributions generated for this study are included in the article/Supplementary Material, further inquiries can be directed to the corresponding author.

## Author Contributions

LB, MI, IN, and SL: study design and experimental work. BD: bioinformatic analysis. LB and MI: writing—original draft preparation. TB: writing—review and editing and project administration. SH, TB, MI, and IN: funding acquisition. All authors have read and agreed to the published version of the manuscript.

## Conflict of Interest

The authors declare that the research was conducted in the absence of any commercial or financial relationships that could be construed as a potential conflict of interest.
