# Supplementary material for: Evaluation of Heterologous Biosynthetic Pathways for Methanol-Based 5-Aminovalerate Production by Thermophilic Bacillus methanolicus
Source: Front Bioeng Biotechnol. 2021 Jun 28;9:686319. doi: 10.3389/fbioe.2021.686319 (PMC8274714; doi:10.3389/fbioe.2021.686319)
Supplement: Supplementary file 1 [file Data_Sheet_1.pdf]

Supplementary material to

Evaluation of heterologous biosynthetic pathways for methanol-based 5-aminovalerate production by thermophilic *Bacillus methanolicus*

Luciana Fernandes Brito[1$], Marta Irla[1$], Ingemar Nærdal[2], Simone Balzer Le[2], Baudoin Delépine[3], Stéphanie Heux[3], Trygve Brautaset[1*]

[$] These authors contributed equally to this work

[1]Department of Biotechnology and Food Sciences, Norwegian University of Science and Technology, Trondheim, Norway
[2]Department of Biotechnology and Nanomedicine, SINTEF Industry, Trondheim, Norway
[3]Toulouse Biotechnology Institute, Université de Toulouse, CNRS, INRA, INSA, Toulouse, France

*Corresponding author: Prof. Dr. Trygve Brautaset
Phone: +47 73593315
Email: trygve.brautaset@ntnu.no

Construction of plasmids used in the study

pBV2xp-*davBA*^{Pp}: The *davBA* operon of *P. putida* KT2440 was PCR amplified from genomic DNA using primers davBA_Pp_F1 and davBA_Pp_R1 generating a 2,567 bp product compatible with assembly cloning together with pBV2xp vector backbone pre-digested with restriction enzymes AflII and SacI. The NEBuilder High-Fidelity DNA Assembly Cloning Kit and protocol as used and led to plasmid pBV2xp-*davBA*^{Pp}.

pBV2xp-*davBA*^{Ws}, pBV2xp-*davBA*Rd, pBV2xp-*davB*^{Ws}-*davA*^{Pc} and pBV2xp-*davA*^{Pc}-*davB*Rd: Four *davBA* operons from alternative hosts were codon-optimized for expression in *B. methanolicus*, synthesised and provided in the pUC57 plasmid from Genscript (Table S1). The alternative hosts were *Williamsia sterculiae*, *Roseobacter denitrificans* and *Parageobacillus caldoxylosilyticus* (*davA* only). The *davBA* operons were subcloned into plasmid pBV2xp by digesting the pUC57 (insert) vectors and pBV2xp digested with AflII and SacI. The *davBA* inserts were separated on agarose gel and purified, and digested pBV2xp was purified using the Qiagen PCR clean up kit. Ligation of inserts into pBV2xp resulted in the four plasmids pBV2xp-*davBA*^{Ws}, pBV2xp-*davBA*Rd, pBV2xp-*davB*^{Ws}-*davA*^{Pc} and pBV2xp-*davA*^{Pc}-*davB*Rd.

Selected *davBA* operons were expressed as single genes using compatible pBV2xp and pMI2mp plasmids for gene expression. The *davB* genes from *P. putida* and *W. sterculiae* were expressed using the xylose-inducible promoter in plasmid pBV2xp, while *davA* gene from *P. caldoxylosilyticus* was constitutively expressed by the *mdh* promoter in pMI2mp.

pBV2xp-*davB*^{Pp}: The *davB*^{Pp} region was amplified from pBV2xp-*davBA*^{Pp} using PCR primers davB_Pp_F1 and davB_Pp_R1 generating a 1,738 bp product with overlapping fragments to the SacI and AflII digested pBV2xp plasmid. Assembly cloning of overlapping fragments resulted in plasmid pBV2xp-*davB*^{Pp}.

pBV2xp-*davB*^{Ws}: The *davB*^{Ws} region was amplified from pBV2xp-*davBA*^{Ws} using PCR primers davB_Ws_F1 and davB_Ws_R1 generating a 1,767 bp product with overlapping fragments to the SacI and AflII digested pBV2xp plasmid. Assembly cloning of overlapping fragments resulted in plasmid pBV2xp-*davB*^{Ws}.

pMI2mp-*davA*^{Pc}: The *davA*^{Pc} region was amplified from plasmid pBV2xp-*davA*^{Pc}-*davB*Rd using PCR primers davA_Pc_F1 and davA_Pc_R1 generating an 879 bp product with overlapping fragments to the SpeI and Acc65I digested pMI2mp plasmid. Assembly cloning of overlapping fragments resulted in plasmid pMI2mp-*davA*^{Pc}.

pMI2mp-*davA*^{Pp}: The *davA*^{Pp} region was amplified from plasmid pBV2xp-*davBA*^{Pp} using PCR primers davA_Pp_F1 and davA_Pp_R1 generating a product with overlapping fragments to the SpeI and Acc65I digested pMI2mp plasmid. Assembly cloning of overlapping fragments resulted in plasmid pMI2mp-*davA*^{Pp}.

pBV2xp-*raiP*^{Ps}: *raiP* gene (1,510 bp) was amplified from genomic DNA of *Peribacillus simplex* with primers raippsfw and raippsrv (Table 1); the plasmid pBV2xp was linearized with restriction enzyme BamHI and overlapping regions were ligated by isothermal DNA assembly method.

pBV2xp-*raiP*^{Sj} and pBV2xp-*raiP*^{Tv}: *raiP* gene from *Scomber japonicus* (1,546 bp) and *Trichoderma viride* (1,910 bp) were codon-optimized for *B. methanolicus* (Table S1) and the synthetic genes were amplified with primers raipsjfw and raipsjrv or raiptvfw and raiptvrv, respectively (Table 1); the plasmid pBV2xp was linearized with restriction enzyme BamHI and overlapping regions were ligated by isothermal DNA assembly method.

pTH1mp-*katA*: *katA* gene was amplified from the genomic DNA of *B. methanolicus* with primers katafw and katarv (Table 1); the plasmid pTH1mp was cut with restriction enzyme PciI and plasmid and PCR product ends were joined by the isothermal DNA assembly method.

pBV2xp-AVA^{Ec} was constructed by amplifying *patA* and *patD* genes from gDNA of *E. coli* with primers as presented in Table 1 and joining the resulting PCR product with SacI and BamHI digested pBV2xp by means of the isothermal DNA assembly method.

pBV2xp-AVA^{Bm} was constructed by amplifying *patA and patD* genes from gDNA of *Bacillus megaterium* with primers as presented in Table 1 and joining the resulting PCR product with SacI and BamHI digested pBV2xp by means of the isothermal DNA assembly method.

pBV2xp-AVA^{Pp} was constructed by amplifying *spuI, spuC, kauB and pauD2* genes from gDNA of *P. putida* with primers as presented in Table 1 and joining the resulting PCR product with SacI and BamHI digested pBV2xp by means of the isothermal DNA assembly method.

pBV2xp-AVA^{Rq} was constructed by amplifying *puo* gene from gDNA of *Rhodococcus qingshengii* and *patD* gene from gDNA of *E. coli* with primers as presented in Table 1 and joining the resulting PCR product with SacI and BamHI digested pBV2xp by means of the isothermal DNA assembly method.

pBV2xp-AVA^{Pa} was constructed by amplifying *puo* gene from gDNA of *Paenarthrobacter aurescens* and *patD* gene from gDNA of *E. coli* with primers as presented in Table 1 and joining

the resulting PCR product with SacI and BamHI digested pBV2xp by means of the isothermal DNA assembly method.

pBV2xp-AVA^{Kr} was constructed by amplifying *puo* gene from gDNA of *K. rosea* and *patD* gene from gDNA of *E. coli* with primers as presented in Table 1 and joining the resulting PCR product with SacI and BamHI digested pBV2xp by means of the isothermal DNA assembly method.

Table S1. Sequences of codon-optimized genes and operons used in this study.

Gene/ operon	Sequence 5’→3’
davBA from *W. sterculiae*	ATGAGAGTTACAACATCAGTTGGAATGGCAGCTAGATCAGTTGATTCAGTTGTTGATACA<br>ACACATAGACCAGTTACAATTTTTGGACCTGATTTTCCGTTTGCATATGATGAATGGTTA<br>GCTCATCCATCTGGAATTGGAACAGTTCCTGCTTCAGCATATGGAACAGAAGTTGCAGTT<br>ATTGGAGCTGGAATGGCAGGAATGACAGCAGCTTATGAACTTATGAGAATGGGATTAAGA<br>CCTGTTGTTTATGAACCGGATAGAATTGGAGGAAGACTTAGATCTGAACCATTTGTTCCT<br>GGAGAACCGGAAGTTGCAGAACTTGGAGGAATGAGATTTCCTGCTTCATCTTCAACATTT<br>TTCAGATATGTTGATCAATTTGGACTTAGAACAAGACCGTTTCCAAATCCTTTAACAGCA<br>GCTGCAGGATCAACAGTTCTTGATCTTAATGGAGAAACACTTTATGCAGAAACATTAGAT<br>GATCTTCCTCCGATTTATAGACAAATTTCTGATGCTTGGGATTCAGCACTTGATAGAATT<br>GCTGGATTTGGAGCATTACAAGATGCTATTAGACATAGAGATGTTGGAGAACTTAAAAAT<br>AGATGGAATCAACTTGTTACAGAATGGGATGATAGATCTTTTTATGATTTTCTTGCAACA<br>TCAAAAGAATTTGGAGAACTTACATTTAGACATAGAGAATTATTTGGACAAGTTGGATTT<br>GGAACAGGTGGATGGGATTCTGATTTTGGAAATTCAATGCTTGAAATTCTTAGAGTTGTT<br>CTTACAAATTGTGATACAGATCAACATCTTATTGTTGGAGGATGCGAACAAGTTCCGAGA<br>TCTCTTTGGACAGATACACCGGCAAGAATTACACATTGGCCAGTTGGAACATCTTTAATG<br>TCACTTCATCATGGAGCTACAAAAGCAGGAGTTGCTGCAATTAGAAGAATTGATGATGAT<br>AGAATTGAAGTTGTTGATAGATATGGAGATTCAAGAGAATTTAGAGCTGTTATTGCAACA<br>TGTCAAGCATGGTTACTTACAACAGAAATTGATTGCGATGAATCTTTATTTTCACAAGAA<br>TTATGGATGGCACTTGATAGAACAAGATATATGCAATCTTCAAAAACATTTGTTATGGTT<br>GATAGACCTTTTTGGAGAGATAGAGATCCGGATACAGGACATGAAACATTATCAATGACA<br>CTTACAGATAGACTTACAAGAGGAACATATCTTTTTGATAATGGACCAAATAGACCTGGA<br>GTTATTTGTTTATCTTATTCATGGATGTCTGATTCACTTAAAATGCTTCCGCATTCTGTT<br>GATAGAAGAGTTGATCTTGCACTTGCTGCATTAAGAAAAATTTATCCAGATGTTGATATT<br>GCTGGACATATTGTTGGAAAACCAATTACAGTTTCATGGGAAGATGATCCTCATTTTCTT<br>GGAGCATTTAAAGGAGCTTTACCAGGACATTATAGATATAATACAAGAATGTATGGACAT<br>TTTGTTCAAGATTCTCTTCCACCTGCAGAAAGAGGAATTTTTATTGCTGGAGATGATGTT<br>TCATTTATGCCTGCATGGGTTGAAGGAGCTGTTCAAACAGGATTAAATGCAGTTTGGGGA<br>GTTATGAGACATGTTGGAGGAGCTACACATCCGGATAATCCGGGACCAGGAGATAGATAT<br>CCGGATATTGGACCACTTGATATTGGATTATAAGCATGCCGATGGATGCAATGCAACAAT<br>TAAGAATTGCTCTTTGGCAATGTCAACCAAGAACAGATGATGTTCCTAGAGCTATTGCAG<br>ATTTAGCAGATAGAGCTTCAGTTGCAGCTGCAGAAGGAGCAAGACTTCTTATTTGCCCTG<br>AAATGTATTTATCAGGATATCATATTGATTCTGCTACAGCAAGAAGACTTGCACAACCGG<br>CTGATGGAGAATGGGCAAGACAAATTGGAGCTGTTGCAAGAGATACAAATATTGCTCTTC<br>TTTATGGATTTCCTGAATTAGCAGGAGATGGAACAGTTTATAATGCTGTTAGACTTGTTG<br>GAAATAATGGAGTTACAGTTGCAGTTCATAGAAAAACACATTTATTTGGAGAAATTGATA<br>GAGCTGCAGTTACAGCATCTGATAGAAGACCAACAGTTTTTCATTTTGAAGGATGGAGAC<br>TTGCACTTCTTATTTGTTATGAAGTTGAATTTCCTGAAGTTGTTAGAAGATTAGCTGTTG<br>CAGGAGCTGATCTTGTTTGCGTTCCTACAGCAAATATGCCGGATTATGATGCTGTTCAAA<br>GAGTTCTTATTCCGGCAAGAGCTTTTGAAAATCAAGTTTATGTTGCATATGCTAATTTTT<br>GTGGAACAGAAAGAGGACTTGATTATGGAGGATTATCACTTATTGCAGGACCATCTGGAG<br>CTACAGTTGTTGAAGCAGGAACAGCTGAAGGAATGATTGTTGGAGATTTAGATAAAGCTG<br>CACTTAGAAGATCAAGACATATGAATCCGTATTTAGCAGAAAGAAGACCAGAACTTTATG<br>ATTAA
davBA from *R. denitrificans*	ATGAAATTAGCACTTTGGCAAACACATCCAAGAACAGGAATTGCAGCTGCATTAACAGCT<br>CTTAGAGAAGCTGCAACATCAGCTGCAACACAAGGAGCAGATGTTCTTATTACACCTGAA<br>ATGTTTGTTGGAGGATATAATATTGGACCGGAAAGAATTGCTACACATGCAGATCATGCT

GCAGAAGTTCTTGATTCACTTACATCTATTGCTAAAACACAAGATATTGCATTAGTTGTT
GGATTAACACTTCCAGCTCCTCCGCTTCCTCATAATGCATGTGTTGTTATTGATAATACA
GGAACACAAGTTGCAAGATATCATAAAACACATCTTTTTGGAGATGTTGATAGAGCACAA
TTTTCAGCAGGAGCTGCATTATCTGAAGTTTTTGATCTTGCTGGATGGAAAGTTGGATTA
GCAATTTGCTATGATGTTGAATTTCCAGAACTTATTAGATCATTAGCTCTTAGAGGAGCA
GAAGTTATTTTAACACCGACAGCTAATATGGAACCATTTGATTCTATTAATACAAGACTT
GTTCCTGCTAGAGCAGAAGAAAATGGAGTTTATGTTGCATATTGTAATTATATTGGAGCT
GAAGCACAATTTACATATAATGGATTATCATGCCTTTCTGGACCGGATGGACAAGATCAT
GTTAGAGCAGAAAAAGCTGAAGCAATGTTATTTGCTACACTTGATAGAGCAGATTTAGCT
AGAGCAAGACAATCTCAAACACATTTACAAGATAGAAGACCTGATCTTTATGGAGATATT
ACATGAAACCAGTTACAGTTTTTGGACCAGATTTTCCTTTTGCATATGATGATTGGATTG
CTCATCCAGATGGATTAGCAACACTTCCTGCAGCTGCACATGGAGCACATGTTGCAATTA
TTGGAGCTGGAGCTGCAGGAGTTATTGCAGGATATGAACTTATGAAACTTGGACTTTGTC
CTATTCTTTTTGAACCGGGACAATTTGGAGGAAGACTTAGATCACAACCTTTTGAAGGAG
CAGAAGGAGTTATTGCTGAACTTGGAGGAATGAGATTTCCGGTTTCATCTACAGGATTTT
ATCATTATGTTGATTTACTTGGAATTCAATCAAAACCATTTCCTAATCCGCTTACACCTG
CTGCAGGATCTACAGTTATTGATTTACTTGGAAAAACATATTATGCACAAACATTACAAG
ATCTTCCTCCGTTATTTCATGAAGTTGCTCAAGCATATGATGCTGCACTTGAACAAGAAG
CTAATTTTTCAGCACTTAAACAAGCTATTAGAGATAGAGATATTCCAAGAATTAAAGAAA
TTTGGAATCCTATTGTTACAGCATGGGATGAAAGAACATTTTATGATTTTGTTGCATCAT
CTGAAGCTTTTAAAAAACTTACATTTCATCATAGAGAAGTTTTTGGACAAGTTGGATTTG
GAACAGGTGGATGGGATTCAGATTTTCCGAATTCTATGCTTGAAATTTTAAGAGTTAATG
TTACAGAATGTGATGATCATCAAAGATATATGGTTGGAGGAGTTGAACAAGTTCCAAGAA
AACTTTGGCAACATAAACCGGATAGATTAGTTCATTGGCCAGCAGGAACATCACTTAGAT
CTTTAAATGATGGAGCAACAAGATCAGGAGCTAAAAGAATTAGAAGATTAGATGCTGGAC
AAATTGAAGTTACAGATGCATGGGGAAGAGCTGAAGGATTTGATGCAGTTCTTGTTACAT
GCCAAACACATTTACTTTCTACACAAATTGATACAGAAGAATCATTATTTTCTCAAGATC
TTTGGATGGCTTTAGATAGAACAAGATATATGCAATCAGCAAAAACATTTGTTATGGTTG
ATAGACCTTTTTGGAAAGATAAACATCCGGTTACAGGAAGAGATACAATGTCTATGACAC
TTACAGATAGAATGACAAGAGGAACATATCTTTTTGATAATGGACCGGATAAACCATCAG
TTATTTGCCTTTCTTATGCATGGATGACAGATGCTCTTAAAGTTTTACCACTTCCTGTTG
AACAAAGAGTTGAATTAGCACTTGCTGCACTTGCTAAAATTTATCCGGATGTTGATATTA
GATCACATATTCTTGGAGATCCAATTACAGTTTCTTGGGAAGCAGATCAAAATTTTCTTG
GAGCATTTAAAGGAGCTTTACCTGGACATTATAGATATAATCATAGAATGTTTGGACATT
TTGTTCAATCAGATATGCCAGCTAGAGAAAGAGGAATTTTTCTTGCAGGAGATGGAGTTT
CATGGACACCTGCATGGGTTGAAGGAGCTGTTCAAACATCTTTAAATGCTGTTGCAGGAA
TTATTGCACATTTTGGAGGAACACCGTCACCAGCTAATCCTTCTCCGCTTGAAGCATATG
AAAAACATGGACCGGTTAGATTATCTGCATAA

davB from
W. sterculiae and
davA from
P. caldoxylosilyticus

ATGAGAGTTACAACATCAGTTGGAATGGCAGCTAGATCAGTTGATTCAGTTGTTGATACA
ACACATAGACCAGTTACAATTTTTGGACCTGATTTTCCGTTTGCATATGATGAATGGTTA
GCTCATCCATCTGGAATTGGAACAGTTCCTGCTTCAGCATATGGAACAGAAGTTGCAGTT
ATTGGAGCTGGAATGGCAGGAATGACAGCAGCTTATGAACTTATGAGAATGGGATTAAGA
CCTGTTGTTTATGAACCGGATAGAATTGGAGGAAGACTTAGATCTGAACCATTTGTTCCT
GGAGAACCGGAAGTTGCAGAACTTGGAGGAATGAGATTTCCTGCTTCATCTTCAACATTT
TTCAGATATGTTGATCAATTTGGACTTAGAACAAGACCGTTTCCAAATCCTTTAACAGCA
GCTGCAGGATCAACAGTTCTTGATCTTAATGGAGAAACACTTTATGCAGAAACATTAGAT
GATCTTCCTCCGATTTATAGACAAATTTCTGATGCTTGGGATTCAGCACTTGATAGAATT
GCTGGATTTGGAGCATTACAAGATGCTATTAGACATAGAGATGTTGGAGAACTTAAAAAT
AGATGGAATCAACTTGTTACAGAATGGGATGATAGATCTTTTTATGATTTTCTTGCAACA
TCAAAAGAATTTGGAGAACTTACATTTAGACATAGAGAATTATTTGGACAAGTTGGATTT
GGAACAGGTGGATGGGATTCTGATTTTGGAAATTCAATGCTTGAAATTCTTAGAGTTGTT
CTTACAAATTGTGATACAGATCAACATCTTATTGTTGGAGGATGCGAACAAGTTCCGAGA
TCTCTTTGGACAGATACACCGGCAAGAATTACACATTGGCCAGTTGGAACATCTTTAATG
TCACTTCATCATGGAGCTACAAAAGCAGGAGTTGCTGCAATTAGAAGAATTGATGATGAT
AGAATTGAAGTTGTTGATAGATATGGAGATTCAAGAGAATTTAGAGCTGTTATTGCAACA
TGTCAAGCATGGTTACTTACAACAGAAATTGATTGCGATGAATCTTTATTTTCACAAGAA
TTATGGATGGCACTTGATAGAACAAGATATATGCAATCTTCAAAAACATTTGTTATGGTT
GATAGACCTTTTTGGAGAGATAGAGATCCGGATACAGGACATGAAACATTATCAATGACA
CTTACAGATAGACTTACAAGAGGAACATATCTTTTTGATAATGGACCAAATAGACCTGGA
GTTATTTGTTTATCTTATTCATGGATGTCTGATTCACTTAAAATGCTTCCGCATTCTGTT
GATAGAAGAGTTGATCTTGCACTTGCTGCATTAAGAAAAATTTATCCAGATGTTGATATT

GCTGGACATATTGTTGGAAAACCAATTACAGTTTCATGGGAAGATGATCCTCATTTTCTT
GGAGCATTTAAAGGAGCTTTACCAGGACATTATAGATATAATACAAGAATGTATGGACAT
TTTGTTCAAGATTCTCTTCCACCTGCAGAAAGAGGAATTTTTATTGCTGGAGATGATGTT
TCATTTATGCCTGCATGGGTTGAAGGAGCTGTTCAAACAGGATTAAATGCAGTTTGGGGA
GTTATGAGACATGTTGGAGGAGCTACACATCCGGATAATCCGGGACCAGGAGATAGATAT
CCGGATATTGGACCACTTGATATTGGATTATAAGCATGCCGATGGAAACATCATATGAAA
TTGCACTTGCTCAAATGACACCTGTTAATTCAGATATTTCTGGAAATCTTGCAAAAATGG
AAGCAATTGCTAATGAATGTAAACAAAAATTTCCGGATGTTAGACTTCTTCTTTTTCCAG
AACTTTGCACAACAGGATATGTTTTATCAGAAACACTTAAAGATGTTGCACAAGCTTGGG
ATGGATTTATTTTTCAAAGAATGTCTAGACTTGCACAAAAACTTCAACTTTATATTGCTT
ATGGATATGTTGAAAAAGATGATGAAGTTAATCTTTATAATTCACTTATTCTTATTCATC
CATCTGGACAATGTGTTGGAAATTATAGAAAAATTCATTTAACACCTCTTGAAAAAGCAT
GGTTTACACCTGGATCAAAACCGGTTTTAGTTGATACAGAACTTGGAAGAATTGGACTTA
TGATTTGTTGGGATTTAGCATTTCCGGAATTAGCTAGATATCTTGCAGTTCATGGAGCTG
AACTTTTACTTGTTCCATGCGCTTGGGAATCTCCATTTCATGAACCTTTTCAAAAATTTG
CAATGGCTAGAGCAATTGATAATACAGTTCATGTTGCAGCTTGCAATCAAGTTGGAAGAT
CATTTCCTTTTCATTTCTTTGGACTTTCATCTATTTATGGACCGGATGGATCTGAAATTG
CTGTTGCAAATATGGATGATCAAGAAGCAATTATTAGAGCTACAATTGATGAAAATTGGA
GACAAGAATTAAAACAAACATTTTATACAATGATGAATGAAAGAAGAACAGATGTTTATT
AA

davA from
P. caldoxylosilyticus
and *davB* from
R. denitrificans

ATGGAAACATCATATGAAATTGCACTTGCTCAAATGACACCTGTTAATTCAGATATTTCT
GGAAATCTTGCAAAAATGGAAGCAATTGCTAATGAATGTAAACAAAAATTTCCGGATGTT
AGACTTCTTCTTTTTCCAGAACTTTGCACAACAGGATATGTTTTATCAGAAACACTTAAA
GATGTTGCACAAGCTTGGGATGGATTTATTTTTCAAAGAATGTCTAGACTTGCACAAAAA
CTTCAACTTTATATTGCTTATGGATATGTTGAAAAAGATGATGAAGTTAATCTTTATAAT
TCACTTATTCTTATTCATCCATCTGGACAATGTGTTGGAAATTATAGAAAAATTCATTTA
ACACCTCTTGAAAAAGCATGGTTTACACCTGGATCAAAACCGGTTTTAGTTGATACAGAA
CTTGGAAGAATTGGACTTATGATTTGTTGGGATTTAGCATTTCCGGAATTAGCTAGATAT
CTTGCAGTTCATGGAGCTGAACTTTTACTTGTTCCATGCGCTTGGGAATCTCCATTTCAT
GAACCTTTTCAAAAATTTGCAATGGCTAGAGCAATTGATAATACAGTTCATGTTGCAGCT
TGCAATCAAGTTGGAAGATCATTTCCTTTTCATTTCTTTGGACTTTCATCTATTTATGGA
CCGGATGGATCTGAAATTGCTGTTGCAAATATGGATGATCAAGAAGCAATTATTAGAGCT
ACAATTGATGAAAATTGGAGACAAGAATTAAAACAAACATTTTATACAATGATGAATGAA
AGAAGAACAGATGTTTATTAAAAAGTAGAGAAGGAGCGATATTAAAATGAAACCAGTTAC
AGTTTTTGGACCAGATTTTCCTTTTGCATATGATGATTGGATTGCTCATCCAGATGGATT
AGCAACACTTCCTGCAGCTGCACATGGAGCACATGTTGCAATTATTGGAGCTGGAGCTGC
AGGAGTTATTGCAGGATATGAACTTATGAAACTTGGACTTTGTCCTATTCTTTTTGAACC
GGGACAATTTGGAGGAAGACTTAGATCACAACCTTTTGAAGGAGCAGAAGGAGTTATTGC
TGAACTTGGAGGAATGAGATTTCCGGTTTCATCTACAGGATTTTATCATTATGTTGATTT
ACTTGGAATTCAATCAAAACCATTTCCTAATCCGCTTACACCTGCTGCAGGATCTACAGT
TATTGATTTACTTGGAAAAACATATTATGCACAAACATTACAAGATCTTCCTCCGTTATT
TCATGAAGTTGCTCAAGCATATGATGCTGCACTTGAACAAGAAGCTAATTTTTCAGCACT
TAAACAAGCTATTAGAGATAGAGATATTCCAAGAATTAAAGAAATTTGGAATCCTATTGT
TACAGCATGGGATGAAAGAACATTTTATGATTTTGTTGCATCATCTGAAGCTTTTAAAAA
ACTTACATTTCATCATAGAGAAGTTTTTGGACAAGTTGGATTTGGAACAGGTGGATGGGA
TTCAGATTTTCCGAATTCTATGCTTGAAATTTTAAGAGTTAATGTTACAGAATGTGATGA
TCATCAAAGATATATGGTTGGAGGAGTTGAACAAGTTCCAAGAAAACTTTGGCAACATAA
ACCGGATAGATTAGTTCATTGGCCAGCAGGAACATCACTTAGATCTTTAAATGATGGAGC
AACAAGATCAGGAGCTAAAAGAATTAGAAGATTAGATGCTGGACAAATTGAAGTTACAGA
TGCATGGGGAAGAGCTGAAGGATTTGATGCAGTTCTTGTTACATGCCAAACACATTTACT
TTCTACACAAATTGATACAGAAGAATCATTATTTTCTCAAGATCTTTGGATGGCTTTAGA
TAGAACAAGATATATGCAATCAGCAAAAACATTTGTTATGGTTGATAGACCTTTTTGGAA
AGATAAACATCCGGTTACAGGAAGAGATACAATGTCTATGACACTTACAGATAGAATGAC
AAGAGGAACATATCTTTTTGATAATGGACCGGATAAACCATCAGTTATTTGCCTTTCTTA
TGCATGGATGACAGATGCTCTTAAAGTTTTACCACTTCCTGTTGAACAAAGAGTTGAATT
AGCACTTGCTGCACTTGCTAAAATTTATCCGGATGTTGATATTAGATCACATATTCTTGG
AGATCCAATTACAGTTTCTTGGGAAGCAGATCAAAATTTTCTTGGAGCATTTAAAGGAGC
TTTACCTGGACATTATAGATATAATCATAGAATGTTTGGACATTTTGTTCAATCAGATAT
GCCAGCTAGAGAAAGAGGAATTTTTCTTGCAGGAGATGGAGTTTCATGGACACCTGCATG
GGTTGAAGGAGCTGTTCAAACATCTTTAAATGCTGTTGCAGGAATTATTGCACATTTTGG
AGGAACACCGTCACCAGCTAATCCTTCTCCGCTTGAAGCATATGAAAAACATGGACCGGT

	TAGATTATCTGCATAA
raiP from *Scomber japonicus*	ATGGAACATTTAGCAGATTGTTTAGAAGATAAAGATTATGATACATTATTACAAACATTAG<br>ATAATGGATTACCACATATTAATACATCTCATCATGTTGTTATTGTTGGAGCAGGAATGGC<br>AGGATTAACAGCAGCAAAATTATTACAAGATGCAGGACATACAGTTACAATTTTAGAAGCA<br>AATGATAGAGTTGGAGGAAGAGTTGAAACATATAGAAATGAAAAAGAAGGATGGTATGCAG<br>AAATGGGAGCAATGAGAATTCCATCTTCTCATAGAATTGTTCAATGGTTTGTTAAAAAATT<br>AGGAGTTGAAATGAATGAATTTGTTATGACAGATGATAATACATTTTATTTAGTTAATGGA<br>GTTAGAGAAAGAACATATGTTGTTCAAGAAAATCCAGATGTTTTAAAATATAATGTTTCTG<br>AATCTGAAAAAGGAATTTCTGCAGATGATTTATTAGATAGAGCATTACAAAAAGTTAAAGA<br>AGAAGTTGAAGCAAATGGATGTAAAGCAGCATTAGAAAAATATGATAGATATTCTGTTAAA<br>GAATATTTAAAAGAAGAAGGAGGATTATCTCCAGGAGCAGTTAGAATGATTGGAGATTTAT<br>TAAATGAACAATCTTTAATGTATACAGCATTATCTGAAATGATTTATGATCAAGCAGATGT<br>TAATGATTCTGTTTCTTATCATGAAGTTACAGGAGGATCTGATTTATTACCAGAAGCATTT<br>TTATCTGTTTTAGATGTTCCAATTTTATTAAATTCTAAAGTTAAACATATTAGACAATCTG<br>ATAAAGGAGTTATTGTTTCTTATCAAACAGGAAATGAATCTTCTTTAATGGATTTATCTGC<br>AGATATTGTTTTAGTTACAACAACAGCAAAAGCAGCATTATTTATTGATTTTGATCCACCA<br>TTATCTATTTCTAAAATGGAAGCATTAAGATCTGTTCATTATGATTCTTCTACAAAAATTT<br>TATTAACATTTAGAGATAAATTTTGGGAAGATGATGGAATTAGAGGAGGAAAATCTATTAC<br>AGATGGACCATCTAGATATATTTATTATCCATCTCATTCTTTTCATACAAATGAAACAATT<br>GGAGTTTTATTAGCATCTTATACATGGTCTGATGAATCTTTATTATTTTTAGGAGCATCTG<br>ATGAAGAATTAAAAGAATTAGCATTAAGAGATTTAGCAAAAATTCATGGAGAACAAGTTTG<br>GGATAAATGTACAGGAGTTATTGTTAAAAAATGGTCTGCAGATCCATATTCTTTAGGAGCA<br>TTTGCATTATTTACACCATATCAACATTTAGAATATGCACAAGAATTATTTTCTTCTGAAG<br>GAAGAGTTCATTTTGCAGGAGAACATACAGCATTTCCACATGCATGGATTGAAACATCTAT<br>GAAATCTGCAATTAGAGCAGCAACAAATATTAATAAAGTTGCAAATGAAGAATCTACAATT<br>GAACATACAAAAGATGAATTATAA
raiP from *Trichoderma viride*	ATGGATAATGTTGATTTTGCAGAATCTGTTAGAACAAGATGGGCAAGAAGATTAATTAGAG<br>AAAAAGTTGCAAAAGAATTAAATATTTTAACAGAAAGATTAGGAGAAGTTCCAGGAATTCC<br>ACCACCAAGAGAAGGAAGATTTTTAGGAGGAGGATATTCTCATGATAATTTACCATCTGAT<br>CCATTATATTCTTCTATTAAACCAGCATTATTAAAAGAAGCACCAAGAGCAGAAGAAGAAT<br>TACCACCAAGAAAAGTTTGTATTGTTGGAGCAGGAGTTTCTGGATTATATATTGCAATGAT<br>TTTAGATGATTTAAAAATTCCAAATTTAACATATGATATTTTTGAATCTTCTTCTAGAACA<br>GGAGGAAGATTATATACACATCATTTTACAGATGCAAAACATGATTATTATGATATTGGAG<br>CAATGAGATATCCAGATATTCCATCTATGAAAAGAACATTTAATTTATTTAAAAGAACAGG<br>AATGCCATTAATTAAATATTATTTAGATGGAGAAAATACACCACAATTATATAATAATCAT<br>TTTTTTGCAAAAGGAGTTGTTGATCCATATATGGTTTCTGTTGCAAATGGAGGAACAGTTC<br>CAGATGATGTTGTTGATTCTGTTGGAGAAAAATTACAACAAGCATTTGGATATTATAAAGA<br>AAAATTAGCAGAAGATTTTGATAAAGGATTTGATGAATTAATGTTAGTTGATGATATGACA<br>ACAAGAGAATATTTAAAAAGAGGAGGACCAAAAGGAGAAGCACCAAAATATGATTTTTTTG<br>CAATTCAATGGATGGAAACACAAAATACAGGAACAAATTTATTTGATCAAGCATTTTCTGA<br>ATCTGTTATTGATTCTTTTGATTTTGATAATCCAACAAAACCAGAATGGTATTGTATTGAA<br>GGAGGAACATCTTTATTAGTTGATGCAATGAAAGAAACATTAGTTCATAAAGTTCAAAATA<br>ATAAAAGAGTTGAAGCAATTTCTATTGATTTAGATGCACCAGATGATGGAAATATGTCTGT<br>TAAAATTGGAGGAAAAGATTATTCTGGATATTCTACAGTTTTTAATACAACAGCATTAGGA<br>TGTTTAGATAGAATGGATTTAAGAGGATTAAATTTACATCCAACACAAGCAGATGCAATTA<br>GATGTTTACATTATGATAATTCTACAAAAGTTGCATTAAAATTTTCTTATCCATGGTGGAT<br>TAAAGATTGTGGAATTACATGTGGAGGAGCAGCATCTACAGATTTACCATTAAGAACATGT<br>GTTTATCCATCTTATAATTTAGGAGATACAGGAGAAGCAGTTTTATTAGCATCTTATACAT<br>GGTCTCAAGATGCAACAAGAATTGGATCTTTAGTTAAAGATGCACCACCACAACCACCAAA<br>AGAAGATGAATTAGTTGAATTAATTTTACAAAATTTAGCAAGATTACATGCAGAACATATG<br>ACATATGAAAAAATTAAAGAAGCATATACAGGAGTTTATCATGCATATTGTTGGGCAAATG<br>ATCCAAATGTTGGAGGAGCATTTGCATTATTTGGACCAGGACAATTTTCTAATTTATATCC<br>ATATTTAATGAGACCAGCAGCAGGAGGAAAATTTCATATTGTTGGAGAAGCATCTTCTGTT<br>CATCATGCATGGATTATTGGATCTTTAGAATCTGCATATACAGCAGTTTATCAATTTTTAT<br>ATAAATATAAAATGTGGGATTATTTAAGATTATTATTAGAAAGATGGCAATATGGATTACA<br>AGAATTAGAAACAGGAAAACATGGAACAGCACATTTACAATTTATTTAGGATCTTTACCA<br>AAAGAATATCAAGTTAAAATTTAA
